# Green Oil‐in‐Water Nanoemulsions for Delivery of Phytochemicals With Pesticidal Activity for Sustainable Food Production and Safety

**DOI:** 10.1111/1541-4337.70455

**Published:** 2026-03-27

**Authors:** Anna Paula Azevedo de Carvalho, David A. Weitz, Carlos Adam Conte‐Junior

**Affiliations:** ^1^ Research Support Group on Nanomaterials, Polymers, and Interaction with Biosystems (BioNano), Department of Biochemistry, Chemistry Institute Federal University of Rio de Janeiro (UFRJ) Rio de Janeiro Rio de Janeiro Brazil; ^2^ Department of Biochemistry, Chemistry Institute, Center for Food Analysis (NAL), Technological Development Support Laboratory (LADETEC) Universidade Federal do Rio de Janeiro (UFRJ) Rio de Janeiro Rio de Janeiro Brazil; ^3^ Graduate Program in Chemistry (PGQu), Institute of Chemistry (IQ) Federal University of Rio de Janeiro (UFRJ), Cidade Universitária Rio de Janeiro Rio de Janeiro Brazil; ^4^ Nanotechnology Network Carlos Chagas Filho Research Support Foundation of the State of Rio de Janeiro (FAPERJ) Rio de Janeiro Rio de Janeiro Brazil; ^5^ Department of Physics, Harvard John A. Paulson School of Engineering and Applied Sciences (SEAS) Harvard University Cambridge Massachusetts USA

**Keywords:** agri‐food systems, antimicrobial activity, crop protection, essential oils, food safety, green nanoformulations, monoterpenes, phytopathogens

## Abstract

Green oil‐in‐water (O/W) nanoemulsions incorporating essential oils (EOs) and food‐derived compounds are gaining prominence as biopesticidal platforms that address the growing demand for sustainable agriculture, food safety, and reduced chemical inputs. These colloidal delivery systems enhance the solubility, stability, and bioefficacy of hydrophobic phytochemicals, offering an environmentally friendly alternative to conventional agrochemicals. This comprehensive review critically examines recent advances in the formulation and application of green O/W nanoemulsions for crop protection. We synthesize evidence on their pesticidal activity against a wide range of pests and phytopathogens relevant to food crop systems, including aphids, insects, fungi, bacteria, and weeds. Emphasis is placed on food‐grade and biodegradable formulation components, such as biosurfactants and natural emulsifiers, as well as their implications for toxicological safety, environmental risk, and scalability. Nanoemulsions have been reported to enhance pest control relative to conventional formulations; however, the reported efficacy varies depending on the formulation composition, target organism, and application conditions. Their full potential remains underexplored in terms of field application, phytotoxicity, and impacts on nontarget organisms. Important gaps persist in addressing underrepresented targets such as plant viruses and nematodes. By integrating concepts of green chemistry, nanotechnology, and food system resilience, this review provides a forward‐looking perspective on the role of green nanoemulsions in sustainable crop management. Their development aligns with the Sustainable Development Goals and offers promising solutions for integrated pest management, organic agriculture, and preharvest food safety, reinforcing the transition toward safer and more resilient food systems.

## 1 Introduction

The increasing demand for agricultural productivity, driven by global population growth, has intensified the use of synthetic pesticides to mitigate yield losses caused by pests and phytopathogens (van den Hoogen et al. 2020). Among the most damaging are nematodes, responsible for up to 10.6% of global soybean production loss, and rust diseases (Kahn et al. 2021), which severely affect grain crops such as coffee, wheat, and soybeans (Fisher et al. 2012). Despite their effectiveness, conventional pesticides—comprising active compounds like organophosphates, carbofuran, and abamectin—are associated with significant environmental and human health risks (Betarbet et al. 2000; Cressey 2015; Jones 2010; Rumschlag et al. 2020; Yan et al. 2016), including carcinogenicity (Cressey 2015) and neurotoxicity (Dowler 2020; Rumschlag et al. 2020). Moreover, intensive pesticide use has contaminated food chains and aquatic environments, contributing to widespread ecological disturbance (Sindhu and Manickavasagan 2023). Regulatory actions have led to the banning of products such as Temik (aldicarb) and Gramoxone (paraquat) in several countries (Paraquat Dichloride; Proposed Interim Mitigation Decision 2016), including Brazil (ANVISA 2015) and European Union (EU) member states (Danny Hakim 2016; European Commission 2019; Lamichhane et al. 2016).

In this context, sustainable agriculture has emerged as a strategic paradigm aligned with the principles of green chemistry, aiming to minimize or eliminate the use and generation of hazardous substances throughout the development and application of agrochemicals (Balter 2011; Fenibo et al. 2021; Perlatti et al. 2014; C. Zhang et al. 2018). The integration of sustainability principles is supported by the UN Sustainable Development Goals (SDGs), particularly SDG 2 (Zero Hunger), SDG 12 (Responsible Consumption and Production), SDG 13 (Climate Action), and SDG 15 (Life on Land), which emphasize the importance of environmental protection, economic viability, and social acceptability in agricultural practices (United Nations 2021). Among the promising alternatives to conventional pesticides (P. Zhang et al. 2021a) are biopesticides**—**plant‐derived compounds (Campos et al. 2018; Chaves et al. 2025; Hay et al. 2020; Pretty 2018) and natural substances with antimicrobial (Carvalho and Conte‐Junior 2021) or pesticidal properties (Eloh et al. 2020; Isman 2020), such as EOs (Diánez et al. 2018), alkaloids, terpenes, and phenolics (Campos et al. 2018; Hay et al. 2020). These phytochemicals exert their effects through specific, often nonlethal mechanisms, offering ecological advantages and lower toxicity to nontarget organisms.

The low water solubility, volatility, and instability of many phytochemicals challenge their effectiveness in field applications. Recent analyses advocate controlled‐release strategies as a way forward to improve pest management outcomes, providing the context within which nano‐enabled delivery systems can be positioned (A. Singh et al. 2024). Colloidal delivery systems, as micro‐/nanoemulsions (NEs), have gained increasing attention for enhancing the solubility, stability, and bioavailability of these compounds while facilitating controlled release and targeted delivery (Fathi et al. 2021; Lelis et al. 2021; Shao et al. 2018).

Several studies have addressed the potential of nanotechnology and nanopesticides in agriculture (Deka et al. 2021; Djiwanti and Kaushik 2019; Hayles et al. 2017; Jampílek and Kráľová 2017; Kah et al. 2013; Luneja and Mkindi 2025), and some recent reviews have explored the potential of NEs in pest management (Feng et al. 2018; Gupta et al. 2024).

However, while previously cited reviews address relevant aspects of nanoformulation for phytochemical delivery, they do not provide a comprehensive synthesis of green O/W NEs composed of naturally derived active and emulsifying agents, nor do they explore their broad‐spectrum efficacy against multiple phytopathogens and pests in the context of sustainable agriculture.

Therefore, this comprehensive review aims to fill a critical gap by providing a comprehensive synthesis of recent advances in green oil‐in‐water (O/W) NEs formulated with essential oils (EOs) and plant‐derived compounds for sustainable food crops. We explore their formulation strategies, physicochemical properties, pesticidal efficacy against key agricultural pests and phytopathogens, and underlying mechanisms of action. In addition, we highlight the potential advantages of these systems over conventional pesticides, with special attention to bio‐based emulsifying agents, challenges in field application and safety, and emerging perspectives for their use as scalable tools in integrated pest management (IPM) and sustainable food production systems.

Accordingly, the following sections progress from the formulation principles and components of green O/W NEs to their mechanisms of action, agricultural applications, and current challenges related to sustainability, scalability, and regulatory considerations.

## 2 Green Oil‐in‐Water Nanoemulsions: Principles and Composition

### 2.1 Structural and Formulation Aspects

**FIGURE 1:**
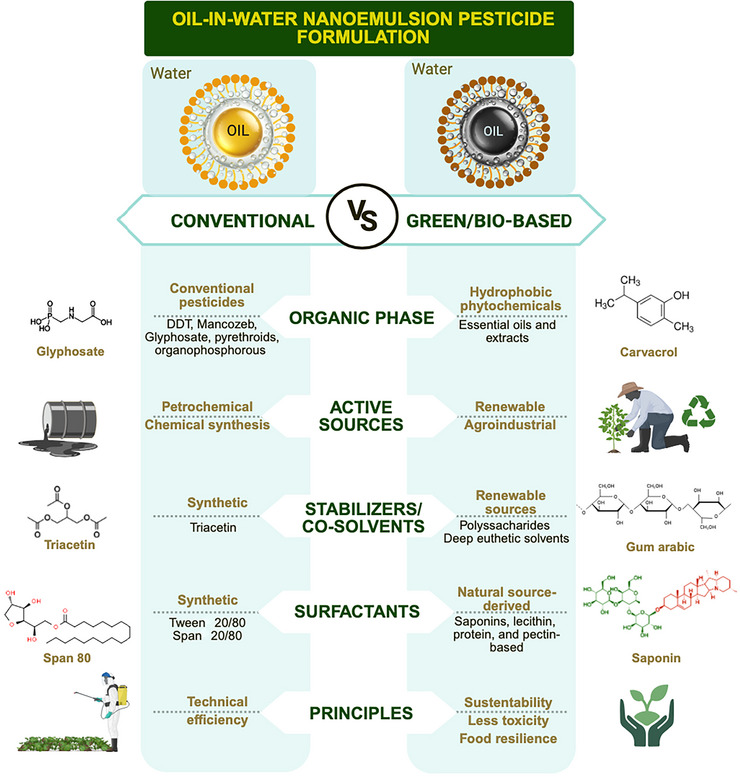
Comparison between conventional and green/bio‐based oil‐in‐water (O/W) nanoemulsion pesticide formulations. Green nanoemulsions incorporate plant‐derived phytochemicals as active ingredients and utilize natural/biodegradable components for emulsification and stabilization, aligning with principles of green chemistry.

Conventional O/W NEs are colloidal systems composed of nanosized oil droplets (< 200 nm) dispersed in water and stabilized by synthetic surfactants and cosolvents to improve the solubility and stability of hydrophobic compounds (McClements 2012). In contrast, green or bio‐based O/W NEs are formulated using renewable, biodegradable, and nontoxic ingredients—such as EOs, plant extracts, natural emulsifiers, and agroindustrial by‐products—designed to align with the principles of green chemistry and reduce environmental impact (Echeverria et al. 2019; Pavoni et al. 2019). These differences in composition, origin, and functional principles between conventional and green NEs are summarized in Figure 1.

Beyond their environmentally friendly composition, green O/W NEs serve as versatile delivery platforms for phytochemicals with pesticidal activity. Depending on the target organism, nanobiopesticides may exhibit insecticidal, larvicidal, acaricidal, bactericidal, fungicidal, virucidal, nematicidal, or herbicidal effects. In insect control, they may additionally act as repellents, ovicides, or adulticides (Gheorghe et al. 2017).

### 2.2 Green Emulsifiers, Solvents, and Pesticides

O/W NEs are colloidal systems composed of nanometric oil droplets (< 200 nm) dispersed in an aqueous continuous phase, in which hydrophobic bioactive compounds—such as EOs or lipophilic phytochemicals—are solubilized within the internal oil phase. These droplets are stabilized at the oil–water interface by emulsifier molecules that reduce interfacial tension and prevent coalescence (Hayles et al. 2017).

Although surfactants such as polysorbates (e.g., Tween 20 and Tween 80) and sorbitan esters (e.g., Span 20 and Span 60) are widely used in food and agrochemical formulations due to their emulsifying efficiency and regulatory acceptance, they are synthetically derived from polyethoxylated sorbitan and fatty acids. Although considered food‐grade, these compounds do not meet the criteria for green chemistry, as they are derived from petroleum and are not readily biodegradable.

Furthermore, the environmental fate of conventional surfactants remains a concern: despite being biodegradable under certain conditions, their interactions with pesticides can unpredictably affect solubility, volatilization, photolysis, and soil mobility. Studies suggest that while surfactants may enhance foliar uptake, their role in root uptake, bioconcentration, and trophic transfer of pesticides in aquatic systems is still poorly understood, underscoring the need for more environmentally benign alternatives (Katagi 2008).

In green or bio‐based formulations, the stabilizing agents are typically natural, biodegradable surfactants or emulsifying systems derived from plant‐based polymers, polysaccharides, or phospholipids (Dammak et al. 2020). Examples include gum arabic, lecithins, saponins, and modified starches, which not only provide interfacial stability but also align with ecotoxicological and regulatory demands for sustainable agrifood systems.

Green NE formulations typically comprise three core components: (i) plant‐based emulsifiers such as saponins, lecithins, and gums; (ii) water as the continuous phase, fulfilling the role of a green solvent; and (iii) natural pesticides including EOs, plant extracts, and neem oil, which provide bioactive compounds with insecticidal, fungicidal, or repellent properties. These components are selected to enhance environmental safety, biodegradability, and performance, while reducing risks to nontarget organisms and the environment, and ensuring compatibility with organic agri‐food products.

## 3 Performance Characteristics

### 3.1 Advantages Over Conventional Systems

These natural systems may also exhibit costabilizing effects, contributing to improved encapsulation efficiency, prolonged shelf life, and enhanced delivery of active ingredients to target pests or pathogens. Compared to conventional pesticide formulations, green NEs offer several key advantages:

- **Physicochemical performance**—High physical stability against droplet aggregation and phase separation (de Castro e Silva et al. 2019a, 2019b; Septiyanti et al. 2020), with stability lasting up to 80 days in some cases (Long et al. 2020), contributing to improved formulation robustness and shelf life.
- **Enhanced biological efficacy**—Higher bioactivity (Long et al. 2020) and increased bioaccessibility of active compounds (R. Zhang et al. 2017; Zhang et al. 2020), as well as stimulation of plant disease resistance mechanisms (M. A. Abdelrasoul et al. 2020), which may allow reduced effective doses compared to free compounds.
- **Controlled delivery and resistance management**—Controlled and targeted release properties (Louni et al. 2018) and potential mitigation of weed and pest resistance development through multitarget phytochemical action (Travlos et al. 2020).
- **Broad‐spectrum pesticidal performance**—Demonstrated efficacy against diverse agricultural targets, including fungi, bacteria, weeds, mites, and insects (Gupta et al. 2024).
- **Environmental compatibility**—Use of biodegradable components and reduced reliance on persistent synthetic pesticides, contributing to lower environmental accumulation and improved ecological sustainability.
- **Reduced nontarget risk and improved applicator safety**—Lower toxicity to nontarget organisms and the environment in many reported cases (Z. Zhang et al. 2021b), together with improved handling and safer foliar application due to elimination of toxic organic solvents (Z. Zhang et al. 2021b).

**FIGURE 2:**
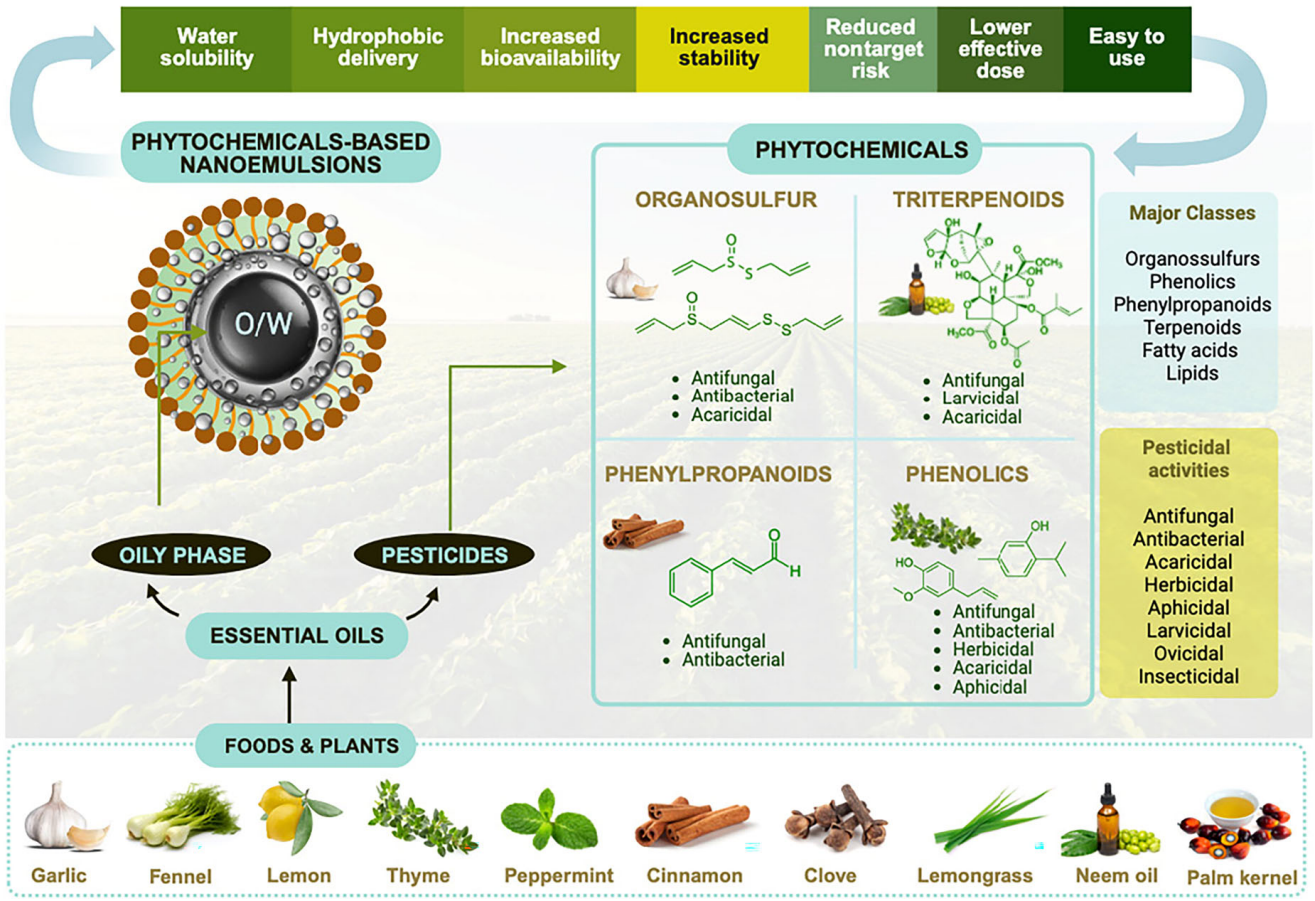
Major phytochemical classes identified in phytochemical‐based oil‐in‐water nanoemulsions, with representative compounds. These natural compounds are associated with distinct pesticidal activities and their potential role in sustainable crop protection.

Although the term “green nanoemulsion” generally refers to formulations composed entirely of natural, renewable, and nontoxic ingredients, it is noteworthy that most studies reviewed still rely on food‐grade synthetic surfactants such as Tween 80 and 20, or Span 60. Therefore, in the context of this review, we adopt a broader definition of green NEs, prioritizing the use of plant‐derived bioactive compounds, while acknowledging that not all formulations fully comply with strict green chemistry criteria for every component. Figure 2 provides an overview of the major phytochemical classes identified in EO‐based NEs, their plant sources, and associated pesticidal activities, illustrating the chemical diversity underpinning their agricultural applications.

### 3.2 Comparison With Other Delivery Systems

In addition to O/W NEs, botanical actives have also been delivered using polymeric nanoparticles/nanocapsules and hydrogel‐based carriers. Compared with solid nanoparticles, O/W NEs are typically simpler to manufacture, readily scalable, and remarkably effective for solubilizing lipophilic EOs, enabling homogeneous foliar application without organic solvents (Kah et al. 2013; McClements 2012; Preeti et al. 2023). In contrast, polymeric nanoparticles or nanocapsules can provide stronger protection of labile actives (e.g., against UV radiation or oxidative degradation) and more programmable release profiles, but they often involve additional carrier materials, more complex processing steps, and potentially higher cost and formulation complexity (Jampílek and Kráľová 2017; Le Dang et al. 2025). Hydrogel‐based matrices can enhance adhesion and moisture retention on plant surfaces, enabling localized or prolonged release (A. Singh et al. 2022). Yet, they can be less suitable for encapsulating highly hydrophobic EOs without coformulation strategies and may face limitations related to viscosity and sprayability in foliar applications (Kaur et al. 2023). Overall, O/W NEs occupy a practical middle ground between performance and deployability, whereas solid carriers and hydrogels may be preferred when maximal protection or long‐term release is required.

### 3.3 Limitations and Trade‐Offs of O/W Nanoemulsions

Despite these advantages, O/W NEs present important limitations, including physical destabilization under field‐relevant stresses (e.g., Ostwald ripening), sensitivity to formulation variables, and potential phytotoxicity when high essential‐oil loadings are used in foliar sprays (Z. Zhang et al. 2021b; Z. Zhang and McClements 2018). Moreover, many reported “green” systems still rely on food‐grade yet synthetically derived emulsifiers (e.g., polysorbates/sorbitan esters), which only partially comply with strict green chemistry principles and may raise concerns regarding environmental fate and regulatory positioning, motivating the development and evaluation of natural emulsifiers (Dammak et al. 2020; Kumari et al. 2018; McClements and Gumus 2016). These constraints underscore the need for biodegradable, bio‐based emulsifiers and the standardized assessment of efficacy–toxicity trade‐offs under realistic agronomic conditions (Guliani et al. 2018).

## 4 Key Factors Governing Phytochemical Delivery

Building on the formulation strategies described above, this section discusses how O/W NEs enhance phytochemical delivery and translate these physicochemical advantages into improved biological performance in crop protection.

### 4.1 Enhanced Solubility and Dispersion

A significant limitation of conventional pesticide formulations is the poor water solubility of many active ingredients, which often necessitates the use of large amounts of toxic organic solvents to achieve effective and uniform application in crops (Hayles et al. 2017). In O/W NEs, surfactant molecules adsorb at the pesticide–water interface, lowering interfacial free energy between the nonpolar (lipophilic) pesticide and the polar aqueous phase. This reduction in interfacial tension promotes uniform dispersion of the active ingredient into nanosized droplets.

Solubilization is further enhanced by the formation of amorphous or less crystalline nanostructures, which inhibit the separation of nonpolar pesticides from the aqueous phase. By preventing droplet coalescence, flocculation, sedimentation, creaming, or Ostwald ripening, NEs maintain formulation stability and preserve pesticidal efficacy over time (Z. Zhang and McClements 2018). In general, nanoemulsification significantly improves the dispersibility of poorly water‐soluble active compounds by reducing droplet size and interfacial tension, allowing for more homogeneous application and eliminating the need for toxic organic solvents. Moreover, nanoemulsification can also enhance the bioefficacy of lipophilic or poorly water‐soluble phytochemicals (McClements 2012).

### 4.2 Protection and Controlled Release

The relevance of controlled‐release and nano‐enabled pesticide formulations for safer and more sustainable crop protection has been extensively discussed in the literature, providing a broader framework within which specific systems, such as NEs, can be positioned (A. Singh et al. 2020). Within this context, the development of targeted nanobiopesticide delivery systems represents a new generation of pesticide formulations designed under ecological principles. These systems typically consist of biodegradable or biopolymeric shells that encapsulate natural, bioactive cores.

Encapsulation protects active ingredients from environmental degradation and enables slow and sustained release, thereby prolonging their persistence under field conditions (Louni et al. 2018). Additionally, encapsulation has been demonstrated to enhance physical stability (Heydari et al. 2020) and improve the aqueous solubility of hydrophobic compounds (Lucia et al. 2020). Improved delivery performance has also been associated with enhanced penetration of active compounds into target tissues (M.‐H. Nguyen et al. 2014, Nguyen et al. 2016), contributing to more effective and environmentally sound applications.

At the nanoscale, particle size enables subcellular delivery, which can increase bioactivity by promoting passive transport across cell membranes (Wang et al. 2012). Controlled release can be achieved by adjusting external stimuli, such as pH, to trigger the release of the active ingredient at specific sites and under defined conditions (Sinha et al. 2017).

In practical applications, nanocapsules can be engineered to release bioactives at predetermined times and locations, thereby improving efficacy while reducing application frequency, dosage, and operational costs (Benelli et al. 2017; Smith et al. 2008). Target‐specific delivery also limits damage to nontarget plant tissues (da Silva Gündel et al. 2019) and mitigates environmental contamination (Srilatha 2011). By enabling kinetic control over release, nanoencapsulation maintains prolonged contact between the active compound and the pest, ensuring sustained efficacy against host‐specific targets (Jerobin et al. 2012; Louni et al. 2018; Sakulku et al. 2009).

Other work reinforces these advantages by demonstrating that NE‐based EO formulations can modulate release kinetics, thereby extending insecticidal activity while reducing phytotoxicity and nontarget effects (Campolo et al. 2020). This example illustrates how controlled‐release behavior can influence formulation performance and environmental safety in specific pest control applications.

### 4.3 Stability Considerations

NEs protect active ingredients from abiotic hydrolysis (Katagi 2002), photo‐degradation (H. M. Nguyen et al. 2013), and oxidative deterioration (Z. Zhang et al. 2021b). The main destabilization pathway is Ostwald ripening, where small droplets dissolve into larger ones over time. Stability can be evaluated by polydispersity index (PDI), with values ≤ 0.3 indicating narrow size distribution, and by zeta potential, where magnitudes near ± 30 mV generally confer stability (Kumari et al. 2018; Zainuddin et al. 2019).

Formulation strategies to enhance stability include optimizing sonication time and emulsifier selection. For instance, *Quillaja* saponin produced thymol NEs with a zeta potential of −31 mV after 50 min, which remained stable over time (Kumari et al. 2018). Similarly, ultrasonication intensity and duration directly influenced droplet size reduction and stability, indicating that extended sonication promoted a narrower size distribution and increased resistance to aggregation (Krittika et al. 2019). Another study emphasized that the type and concentration of emulsifier determine not only droplet size but also shelf stability, reporting that optimized polysorbate‐based NEs maintained physicochemical stability for several weeks without phase separation (Lucia et al. 2020).

Other stabilization strategies involve polymer–surfactant coadsorption, which can arrest Ostwald ripening (Chebil et al. 2013), or blending medium‐chain triglycerides with natural emulsifiers to suppress droplet growth during storage (Chang et al. 2015; Z. Zhang et al. 2021b).

### 4.4 Bioavailability and Bioactivity Improvement

The biological performance of NEs often improves due to increased bioavailability and cellular uptake of pesticidal compounds (Mustafa and Hussein 2020). Bioactivity is strongly influenced by droplet size (< 200 nm), particle size distribution, and surface tension. Smaller droplets with narrow distribution generally result in higher pesticidal efficacy (Anjali et al. 2012; Xu et al. 2010). Surfactant selection plays a crucial role; for example, Tween 80 tends to produce the smallest droplets in spontaneous emulsification, thereby enhancing dispersion and penetration (S. Pandey et al. 2020).

This enhanced bioactivity is attributed to a greater surface area for contact and improved penetration through waxy plant cuticles and insect exoskeletons, as well as better wetting and spreading on plant surfaces (Guan et al. 2018; Hayles et al. 2017; Katagi 2008). As an example, garlic EO NEs (52.7 nm) reduced the minimum inhibitory concentration (MIC) against *Penicillium italicum* from 3.7% to 0.01265%, representing an approximately 300‐fold increase in potency (Long et al. 2020).

In some cases, factors such as surfactant and cosurfactant composition have a greater impact on efficacy than droplet size. Zeng et al. (2019) demonstrated that the optimal hydrophilic–lipophilic balance, achieved with Cremophor EL/triacetin and butanol, significantly improved the insecticidal activity of norcanthridin NEs against *Plutella xylostella*, likely by altering interfacial tension and slowing active compound diffusion (Zeng et al. 2019).

Similarly, Krittika et al. (2019) highlighted that NEs stabilized with biocompatible surfactants promoted superior bioaccessibility of lipophilic compounds compared to conventional emulsions, indicating that the interfacial composition is as critical as droplet size for enhancing bioavailability. Lucia et al. (2020) further supported these findings by demonstrating that the nanoscale dimension of EO‐based formulations enhanced penetration and uptake in target organisms, resulting in more consistent biological responses under realistic exposure conditions. Together, these studies emphasize that NE‐mediated improvements in bioavailability arise from the interplay between droplet size, surfactant chemistry, and interfacial dynamics rather than a single parameter alone.

### 4.5 Impact on Phytochemical Profile

**FIGURE 3:**
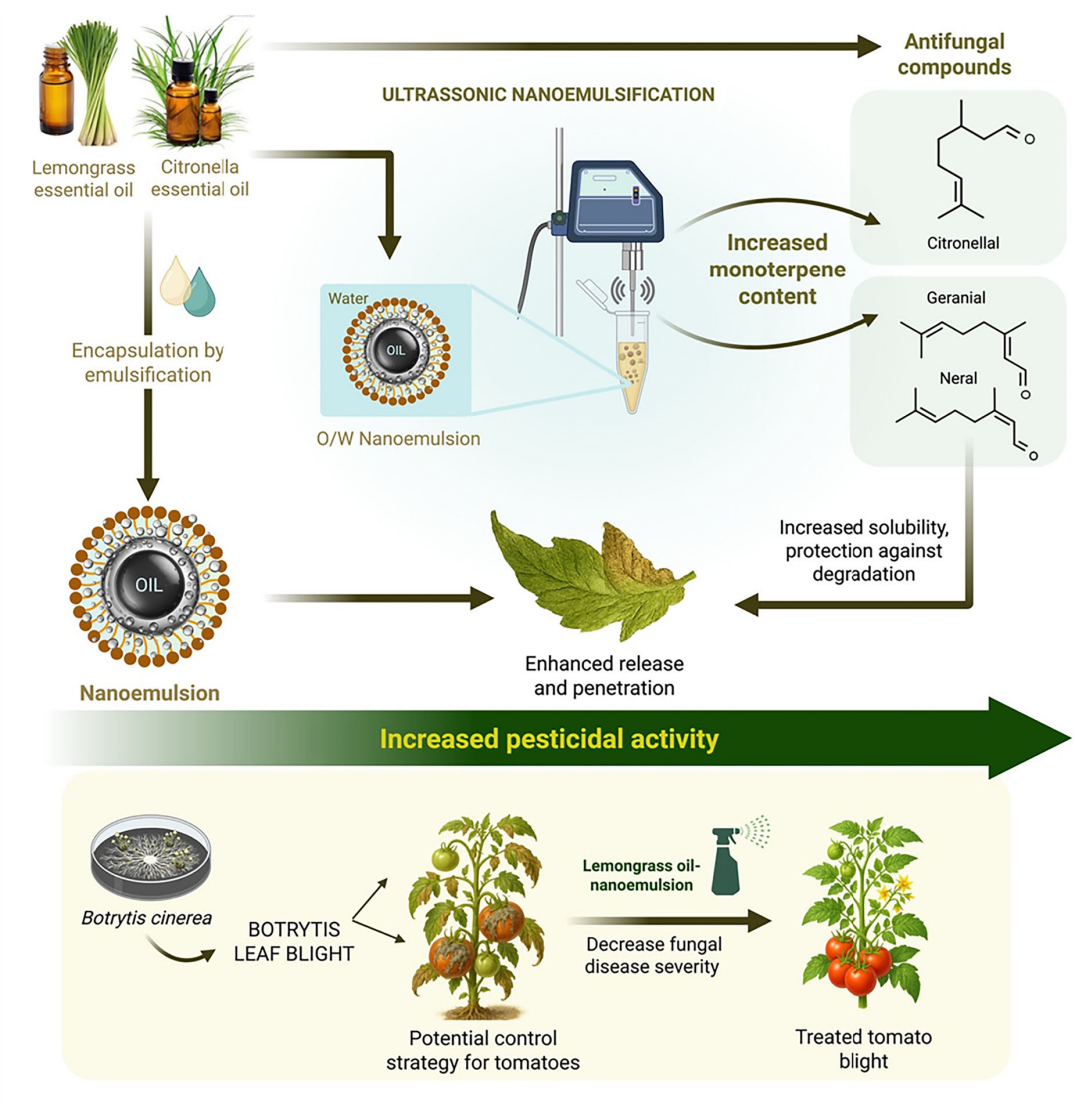
Ultrasound‐assisted essential oil nanoemulsion synthesis increased the monoterpene contents and bioactivity against *Botrytis* fungal disease compared to the original EO—illustrative example of how this nanoemulsion‐based strategy could be applied to manage *Botrytis cinerea* in tomato crops.

EOs are complex mixtures of phytochemicals, predominantly low‐molecular‐weight terpenoids and phenylpropanoids, which confer characteristic biological activities. When formulated into biopesticidal O/W NEs, the chemical profile of these oils can be altered, potentially enhancing their bioactivity. For example, in lemongrass (*Cymbopogon citratus*) and citronella (*Cymbopogon nardus*) EOs, the concentrations of key active constituents—neral and geranial in lemongrass, and citronellal in citronella—were found to be higher in the NE form compared to the pure oils (Hassanin et al. 2018). The authors attributed these changes to sonochemical effects occurring during NE synthesis, in which acoustic cavitation may disrupt chemical bonds and promote atomic rearrangements around donor atoms, thereby modifying the molecular composition (Hassanin et al. 2018). Although the study focused on Botrytis infection in poinsettia, the findings may be translatable to tomato crops, a significant food plant highly affected by gray mold. Thus, Figure 3 shows an example of how the NE‐based strategy could be applied to manage Botrytis cinerea in tomato plants.

## 5 Applications in Sustainable Food Crops

Green O/W NEs have been widely explored as delivery systems for phytochemicals with pesticidal properties, targeting phytopathogenic bacteria, fungi, spider mites, insects, aphids, and weeds. The active compounds, typically secondary metabolites from EOs or plant extracts, are encapsulated in nanosized droplets using green, nontoxic emulsifiers—most commonly polysorbate‐type nonionic surfactants. Formulations are produced through high‐energy (ultrasonication, high‐pressure homogenization) or low‐energy emulsification methods, selected based on the physicochemical characteristics of the active ingredient and the desired field performance. A summary of representative formulations and their target pests is provided in Table 1.

**TABLE 1:** Summary of O/W nanoemulsions as delivery systems for phytochemicals purposing biopesticidal activity.

Active ingredient	Plant species derived	Processing method	Emulsifying system	Continuous phase	Bioactivity (target pest)	Refs.
Neem oil	*Azadirachta indica*	Ultrasonication	Tween 20	Water	Larvicidal (*C. quinquefasciatus*)	Anjali et al. (2012)
Eucalyptus (EO)	*Eucalyptus globulus*	Low‐energy emulsification	Tween 80	Distilled water	Insecticide (*T. castaneum*)	Pant et al. (2014)
				Karanja and jatropha aqueous extract		
Asteraceae plants (EO)	*Ageratum conyzoides*	High‐pressure homogenization	n/r	Water	Insecticide: ovicidal and adulticidal (*C. maculatus*)	Nenaah et al. (2015)
Pyrethrum Daisy flower	*Tanacetum cinerariifolium*	EIP	Tween 20 Tween 80	Water/glycerol	Insecticide: ovicidal (*A. gossypii*)	Kalaitzaki et al. (2015)
Lemon EO	*Citrus limon*					
Eugenol oil	n/a	Ultrasonication	Tween 20	Water	Antifungal (*F. oxysporum* f. sp. *Vasinfectum*)	Abd‐Elsalam and Khokhlov (2015)
Wild thyme EO	*Thymus capitatus*	Ultrasonication	Tween 20 Tween 80/chloroform^*^	Water	Herbicidal (*C. arvensis*)	Balah and Abd El Azim (2016)
Cultivated thyme EO	*Thymus capitatus*					
Marjoram EO	*Majoran hortensis*					
Neem oil with citronella oil—0.5%–05%	*Azadirachta indica*	Spontaneous emulsification	Tween 20	Water	Antifungal (*R. solani* and *S. rolfsii*)	Ali et al. (2017)
Citronella oil with neem oil—0.50%–5%	*Cymbopogon nardus*		Triton‐X100			
Sweet basil EO	*Ocimum basilicum*	Ultrasonication	Tween 80	Water	Antifungal (*Fusarim oxysporum*)	Hassanin, Abd‐El‐Sayed, et al. (2017)
Marjoram EO	*Majoran hortensis*					
Peppermint EO	*Mentha piperita*					
Spearmint EO	*Mentha spicata*					
Thyme EO	*Thymus vulgaris*					
Thyme EO	*Thymus vulgaris*	Ultrasonication	Tween 80	Water	Antifungal (*S. sclerotiorum*)	Hassanin et al. (2017)
Lemongrass EO	*Cymbopogon citratus*	Ultrasonication	Tween 80	Water	Antifungal (*B. cinerea*)	Hassanin et al. (2018)
Citronella EO	*Cymbopogon nardus*	Ultrasonication	Tween 80	Water	Antifungal (*B. cinerea*)	Hassanin et al. (2018)
Horsemint EO	*Mentha longifolia*	High‐pressure homogenization	Tween 20	Water	Insecticidal (*E. kuehniella*)	Louni et al. (2018)
Thymol EO	*Thymus vulgaris*	Ultrasonication	*Quillaja* tree—saponin	Water	Antibacterial (*X. axonopodis* pv. *Glycine*)	Kumari et al. (2018)
Weeping bottlebrush EO	*Callistemon viminalis*	Ultrasonication	Tween 80	Water	Acaricidal (*T. hassani*)	Badawy et al. (2018)
Oregano EO	*Origanum vulgare*					
R‐limonene (synthetic)	n/a					
Pulegone (synthetic)	n/a					
Garlic EO	*Allium sativum*	Ultrasonication	Tween 20	Water	Acaricidal (*A. oleae* and *T. hassani*)	Mossa et al. (2018)
Clove EO and lemongrass EO	*Syzygium aromaticum* and *C. citratus*	Low‐energy emulsification	Tween 20 and Castor oil	Water	Antifungal (*F. oxysporum*)	Sharma et al. (2018)
Sweet flag oil	*Acorus calamus*	Spontaneous emulsification	Tween	Water	Insecticidal against rice weevil (*S. oryzae*)	Dhivya et al. (2019a)
Sweet flag oil	*Acorus calamus*	Ultrasonication	Tween 80/span 80	Water	Insecticidal against pulse beetle (*C. maculatus*)	Dhivya et al. (2019b)
Cinnamon EO	*Cinnamomum verum*	Ultrasonication	Tween 80	Water	Fungicidal against several fungal types	Miastkowska et al. (2020)
Thyme EO	*Thymus vulgaris*					
Manuka EO	*Leptospermum scoparium*					
Tea tree EO	*Camelia sinensis*					
Cinnamon EO	*Cinnamomum verum*	PIC	Tween 80	Water	Fungicidal against several phytopathogenic fungi	Miastkowska et al. (2020)
Thyme EO	*Thymus vulgaris*					
Manuka EO	*Leptospermum scoparium*					
Tea tree EO	*Camellia sinensis*					
Eucalyptus (EO)	*Eucalyptus globulus*	High‐pressure homogenization	Tween 80	Water	Insecticidal (*T. castaneum* and *S. oryzae* of rice)	Adak et al. (2020)
Monoterpenes (synthetic)	n/a	Ultrasonication	Tween 80	Water	Antibacterial (*P. carotovorum* and *R. solanacearum*)	M. A. Abdelrasoul et al. (2020)
Eugenol (synthetic)	—	Spontaneous emulsification	Tween 80/span 80	Water	Antigungal (*G. cingulata*)	da Silva Gündel et al. (2019)
Eugenol oil	n/a	Spontaneous emulsification	Tween 80/span 80	Water	Antigungal (*G. cingulate*)	Velho et al. (2019)
Neem oil	*Azadirachta indica*	Ultrasonication	Tween 20	Water	Antifungal (*A. niger* and *P. citrinum*)	de Castro e Silva et al. (2019a)^a^
Eugenol oil	*Syzygium aromaticum*	Spontaneous emulsification	Polysorbate 80 plus Span 80	Water	Fungicide; Ecosafety (*Folsomia candida*)	da Silva Gündel et al. (2019)
Carrot grass (crude extract)	*Parthenium hysterophorus*	High‐pressure homogenization	Tensiofix: Tween 80	Water	Herbicidal (*Diodia ocimifolia*)	Zainuddin et al. (2019)
Palm kernel oil ester	n/a					
EO: Basil, cinnamon, eucalyptus, mandarin, oregano, peppermint, tea tree, and thyme	n/a	Microfluidization		Water	Insecticidal against rice weevil (*S. oryzae*)	Hossain et al. (2019)
Cinnamon EO	*Cinnamomum verum*	Ultrasonication^c^	Tween 80	Water	Antifungal (*A. niger, R. arrhizus, Penicillium* sp., and *C. gloeosporioides*)	Pongsumpun et al. (2020)
Garlic oil (synthetic)	*Allium sativum*	Ultrasonication	Tween 80 plus Span 80	Water	Antifungal (*P. italicum*)	Long et al. (2020)
Peppermint oil	*Mentha piperita*	Ultrasonication	Tween 80	Water	Fungicide (*A. solani*)	S. Pandey et al. (2020)
Tasmanian blue gum EO	*Eucalyptus globulus*	High‐pressure homogenization	Tween 80 plus gum Arabic	Water	Insecticidal ‐adulticidal, ovicidal, and repellent (*C. maculatus*)	Ya‐Ali et al. (2020)
			Tween 80 plus Span 80			
Carvacrol EO	n/a	Microfluidization (10,000–20,000 psi)	*Quillaja* saponin/MCT oil (1:1)	Water	Antimicrobial	Z. Zhang et al. (2021b)

EO: essential oil; O/W NEs: oil‐in‐water nanoemulsion; EIP: emulsion inversion point; SDBS: sodium dodecylbenzene sulfonate (anionic surfactant); n/r: not reported; n/a: not applicable; PDI: polydispersity index; PIC: phase inversion composition; *: chloroform as cosurfactant (10:1 surfactant/cosurfactant); ^c^: At optimum condition: sonication time of 266 s, 4.82°C, and Tween 80 of 3% w/w; MCT: medium‐chain triglyceride.

Beyond conceptual advantages, several studies reviewed here provide quantitative evidence supporting the contribution of O/W NEs to sustainable food production and safety. For instance, garlic EO NEs reduced the MIC against *Penicillium italicum* from 3.7% to 0.01265%, representing an approximately 300‐fold increase in antifungal potency compared to the free oil, which enables a substantial dose reduction (Long et al. 2020). Similarly, lemongrass and citronella EO NEs achieved complete inhibition of *Botrytis cinerea* in vitro and significantly reduced disease severity in plants, reaching efficacy levels comparable to or exceeding those of carbendazim‐based formulations, while relying on plant‐derived actives (Hassanin et al. 2018). In another example, thymol NEs stabilized with Quillaja saponin exhibited a MIC of 0.5 mg/mL against *Xanthomonas axonopodis* and promoted soybean growth under greenhouse conditions, suggesting both pesticidal efficacy and potential stimulation of plant defense mechanisms (Kumari et al. 2018). Collectively, these quantitative outcomes demonstrate that NE‐based delivery can enhance bioefficacy, reduce application doses, and lower chemical inputs, thereby supporting sustainable crop protection and improved food safety.

## 6 Biopesticidal Efficacy of Green O/W Nanoemulsions

Building upon their physicochemical advantages and potential for sustainable agriculture, an increasing number of studies have investigated the biopesticidal efficacy of green O/W NEs. The following section examines how these systems combat various crop pests and pathogens, including bacteria, fungi, weeds, mites, and insects. Given the breadth of studies available, this section prioritizes a comprehensive synthesis of the literature, while detailed experimental parameters are primarily summarized in the tables for clarity and completeness.

### 6.1 Antimicrobial Activity

#### 6.1.1 Antibacterial Activity

Plant pathogenic bacteria, such as *X. axonopodis* (Kumari et al. 2018), *Pectobacterium carotovorum* (syn. *Erwinia carotovora*), and *Ralstonia solanacearum* (M. A. Abdelrasoul et al. 2020; Marei et al. 2018), cause significant yield losses in economically important crops, including soybeans and potatoes. Green O/W NEs carrying EOs or isolated monoterpenes have been investigated as antibacterial agents, offering improved solubility, stability, and bioavailability compared to free compounds. However, concentration thresholds must be considered to avoid phytotoxicity in foliar applications.

The report of Zhang et al. (2021b) highlighted the importance of dosage control when applying EO‐based NEs to foliage. In spinach leaves, foliar spraying with carvacrol NEs at 5% caused marked phytotoxic effects, which were not observed at lower concentrations. The authors concluded that the EO component, rather than other formulation ingredients, was responsible for the damage, establishing a critical threshold for safe agricultural use (Z. Zhang et al. 2021b).

Thymol NEs were stabilized with *Quillaja* saponin (droplet size ≈ 80–100 nm, PDI ≈ 0.2) and demonstrated strong antibacterial activity against *X. axonopodis*, the causal agent of bacterial pustule in soybean. The formulation exhibited a MIC of 0.5 mg/mL and, interestingly, promoted soybean plant growth under greenhouse conditions. The authors attributed these effects to both direct bactericidal action and potential stimulation of plant defense (Kumari et al. 2018).

Hybrid citral oil–chitosan NEs were made via ultrasonication, obtaining droplets of 27–1283 nm (PDI 0.51–0.61). Against *E. carotovora*, the NEs displayed enhanced antibacterial activity compared to pure citral, as shown by larger inhibition zones in vitro. The chitosan component was proposed to contribute through cell wall disruption and chelation of essential nutrients for bacterial growth (Marei et al. 2018).

Abdelrasoul et al. (2020) reported cinnamaldehyde NEs (5% oil phase) with superior in vitro activity against *P. carotovorum* (MIC = 60 mg/L) and *R. solanacearum* (MIC = 100 mg/L) compared to the free monoterpene. In vivo assays on *R. solanacearum‐infected* potato leaves confirmed protection against soft rot and brown rot, accompanied by increased activities of peroxidase (POD), polyphenol oxidase (PPO), and higher total phenolic content. The authors proposed that these biochemical changes reflected lignin deposition and the induction of defense‐related enzymes, strengthening the cell wall against pathogen invasion (M. A. Abdelrasoul et al. 2020). The in vitro (M. A. Abdelrasoul et al. 2020; Marei et al. 2018) and in vivo (M. A. Abdelrasoul et al. 2020) antibacterial activity was significantly enhanced by NE compared to the pure monoterpenes. Moreover, these compounds act as host resistance mechanisms, blocking bacterial invasion (Lyon and McGill 1989).

An earlier study compared NEs of five monoterpenes—carvone, cinnamaldehyde, citral, geraniol, and pulegone—against *P. carotovorum* and *R. solanacearum*. Among the tested compounds, cinnamaldehyde NE showed the highest antibacterial efficacy, followed by citral, while pulegone exhibited the weakest activity. The study reinforced that NE formulation markedly enhances the bactericidal effect of hydrophobic plant metabolites (M. Abdelrasoul et al. 2018).

#### 6.1.2 Antifungal Activity

Fungal phytopathogens cause substantial yield and quality losses across many crops. They can be soilborne, seed‐borne, or airborne, and their management must preserve nontarget mycoflora that contribute to soil fertility and ecological balance (Jampílek and Kráľová 2017; Miastkowska et al. 2020). Green O/W NEs prepared with EOs or plant extracts have emerged as promising alternatives to synthetic fungicides, offering high antifungal efficacy with lower environmental impact.

The studies reviewed addressed diverse fungal pathogens, including Fusarium oxysporum (Abd‐Elsalam and Khokhlov 2015; Hassanin, Abd‐El‐Sayed, et al. 2017; Sharma et al. 2018), Botrytis cinerea (Hassanin et al. 2018), Sclerotinia sclerotiorum (Hassanin et al. 2017), Sclerotium rolfsii (Ali et al. 2017), Rhizoctonia solani (Ali et al. 2017; Feng et al. 2020), Pyricularia oryzae and Phomopsis amygdali (Feng et al. 2020), Colletotrichum gloeosporioides (Feng et al. 2020; Pongsumpun et al. 2020) or *Glomerella cingulate* (Velho et al. 2019), Penicillium italicum and Rhizopus stolonifer (Long et al. 2020), Aspergillus flavus (de Castro e Silva et al. 2019a, 2019b), Penicillium citrinum (de Castro e Silva et al. 2019a, 2019b), Aspergillus niger (Marei et al. 2018; Pongsumpun et al. 2020), *Rhizopus arrhizus* (Pongsumpun et al. 2020). Details for all studies are provided in Table 2; herein, we highlight representative examples that illustrate key formulation–efficacy relationships.

**TABLE 2:** Antimicrobial activity of green O/W nanoemulsions against bacteria and fungi associated with crop diseases—Evidence from in vitro and greenhouse studies.

Antibacterial activity										
Biopesticide			O/W NE properties			Antibacterial activity				
Active ingredient	Plant species derived	Main phytochemicals	Mean droplet size	PDI value	Zeta potential	Plant disease	Target pest (bacteria)	Inhibition	In vitro/greenhouse	Refs.
Thymol (0.06% v/v)^f^	n/a	Thymol > 98.5%	274.7 nm	0.13	−32.66 mV	Pustule disease in soybeans	*Xanthomonas axonopodis*	I: 100%;	In vitro	Kumari et al. (2018)
								PEDC: 95%^g^	Soybean seeds	
Citral oil (0.04–0.32g)	n/a	Geranial (Trans, 55%–70%) and Neral (Cis, 35%–45%)	27–1283 nm	0.508–0.614	—	Bacteria of blackleg	*Erwinia carotovora*	EC_50_ = 23 mg/L	In vitro	Marei et al. (2018)
Monoterpenes (5%)^g^	n/a	(R)‐carvone 98%	67 nm	0.177	+0.816 mV	Bacterial Soft Rot	*Pectobacterium carotovorum*	MIC:400 mg/L	In vitro; and on Spunta potato	M. A. Abdelrasoul et al. (2020)
		Cinnamaldehyde 98%	128 nm	0.322	−0.052 mV			MIC:60 mg/L		
		Citral 95%	56.6 nm	0.130	−0.216 mV			MIC:100 mg/L		
		Geraniol 98%	176 nm	0.630	−1.110 mV			MIC:160 mg/L		
		Pulegone 92%	58.8 nm	0.311	−0.572 mV			MIC:200 mg/L		
		(R)‐carvone 98%	67 nm	0.177	+0.816 mV	Bacterial Wilt	*Ralstonia solanacearum*	MIC:500 mg/L	In vitro; and on Spunta potato	M. A. Abdelrasoul et al. (2020)
		Cinnamaldehyde 98%	128 nm	0.322	−0.052 mV			MIC:100 mg/L		
		Citral 95%	56.6 nm	0.130	−0.216 mV			MIC:170 mg/L		
		Geraniol 98%	176 nm	0.630	−1.110 mV			MIC:300 mg/L		
		Pulegone 92%	58.8 nm	0.311	−0.572 mV			MIC:600 mg/L		

**Antifungal activity**										
**Biopesticide**			**O/W NE properties**			**Antifungal activity**				
**Active ingredient in NE**	**Plant species derived**	**Main phytochemicals**	**Mean droplet size**	**PDI value**	**Zeta potential**	**Plant disease**	**Target pest (fungi)**	**Inhibition**	**Assay/crop tested**	**Refs**.
Sweet basil EO	*Ocimum basilicum*	Linalool	23 nm	0.244	—	Fusarium wilt	*Fusarium oxysporum*	I: 90%^e^	In vitro; cumin seeds^d^	Hassanin, Abd‐El‐Sayed, et al. (2017)
Marjoram EO	*Majorana hortensis*	Terpinen‐4‐ol	46 nm	0.125	—			I: 49%^e^		
Peppermint EO	*Mentha piperita*	Menthol, Menthone	74 nm	0.436	—			I: 77%^e^		
Spearmint EO	*Mentha spicata*	Carvone	59 nm	0.181	—			I: 37%^e^		
Thyme EO	*Thymus vulgaris*	p‐cymene, thymol	65 nm	0.165	—			I: 100%^e^		
Thyme EO—1%	*Thymus vulgaris*	p‐cymene, thymol	34 nm	—	—	White mold disease	*Sclerotinia sclerotiorum*	I: 100%	In vitro; Fennel seeds	Hassanin et al. (2017)
Lemongrass EO—8000 ppm	*Cymbopogon citratus*	Neral and geranial	66 nm	0.183	—	Botrytis blight	*Botrytis cinerea*	I: 100%	In vitro; Poinsettia^d^	Hassanin et al. (2018)
Citronella EO—8000 ppm	*Cymbopogon nardus*	Citronella, linalool, citronelol	51 nm	0.227	—	Botrytis blight	*Botrytis cinerea*	I: 100%		
Clove EO/lemongrass EO	*Syzygium aromaticum/C. citratus*	CEO: eugenol, β‐caryophyllene LEO: geranial, neral	77 nm	0.207	—	Fusarium wilt	*Fusarium oxysporum*	MIC: 4000 mg/L; I: 76%	Tomato	Sharma et al. (2018)
Eugenol oil—15.5 mg/mL	n/a	Eugenol	191 nm	0.28	−8.9 mV	Anthracnose	*G. cingulata*	MIC: 80 µg/mL	In vitro	Velho et al. (2019)
Neem oil—3% (w/v)	*Azadirachta indica*	Azadirachtin	59 nm	0.2	—	Kernel Rots	*Aspergillus flavus*	IZ: 28 mm	In vitro; Soybean seeds	de Castro e Silva et al. (2019a, 2019b)
						Yellow rice disease	*Penicillium citrinum*	IZ: 25 mm		
Manuka oil (1.5% by ultrasound)	*Lystospermum scoparium*	Sesquiterpenoids	98 nm (*t* = 0) and 146 nm (*t* = 24 h)	0.427 (*t* = 0) and 0.450 (*t* = 24)	—	Several fungal diseases	Fungicidal against. several fungi types	I: 100%	In vitro	Miastkowska et al. (2020)
Eugenol oil—2%	n/a	Eugenol, phenolics	80 nm	—	—	Fusarium wilt of cotton	*Fusarium oxysporum* f. sp. *vasinfectum*	IZ: 5 cm	In vitro, Cottonseeds	Abd‐Elsalam and Khokhlov (2015)
Garlic oil—5.5%	*Allium sativum*	Mainly: organosulfur	52.27 nm	0.271 after 80 days	—	Blue mold disease	*Penicillium italicum*	MIC: 0.01265%	In vitro	Long et al. (2020)
Citral oil (0.04–0.32g)	n/a	Geranial (Trans, 55%–70%) and Neral (Cis, 35%–45%)	27–1283 nm	0.508–0.614	—	black mold (or black rot)	*Aspergillus niger*	EC_50_ = 278 mg/L	In vitro	Marei et al. (2018)
						Head root disease	*Rhizopus stolonifer*	EC_50_ = 221 mg/L	In vitro	
Neem oil—10% (plus citronella oil—5%)	*Azadirachta indica*	Azadirachtin	17.80 nm	—	—	Rice sheath blight	*Rhizoctonia solani*	ED_50_ = 13.67 mg/L	In vitro	Ali et al. (2017)
						Southern blight	*Sclerotium rolfsii*	ED_50_ = 14.71 mg/L		
Citronella oil—10% (plus neem oil—5%)	*Cymbopogon nardus*	Citronella, linalool, citronelol	8.12–12.04 nm	—	—	Rice sheath blight	*Rhizoctonia solani*	ED_50_ = 25.64 mg/L		
						Southern blight	*Sclerotium rolfsii*	ED_50_ = 20.88 mg/L		
Peppermint oil—1%	*Mentha piperita*	Mainly monoterpenes	20–40 nm	—	−16.2 mV	Early blight disease	*Alternaria solani*	I: up to 80%	Tomato^d^	A. Pandey et al. (2021)
Cinnamon EO—1% w/w	*Cinnamomum zeylanicum*	trans‐cinnamaldehyde	66 nm	0.15	—	Sooty mold	*Aspergillus niger*	IZ: 23.74 mm	In vitro	Pongsumpun et al. (2020)
						Soft rot	*Rhizopus arrhizus*	IZ:22.12 mm		
						Postharvest fruit spoilage	*Penicillium* spp.	IZ:19.27 mm		
						Anthracnose	*Colletortrichum gloeosporioides*	IZ:16.34 mm		
D‐limonene—10% (synthetic)	n/a	D‐limonene	112 nm	—	—	Rice blast	*Pyricularia oryzae*	I:48.7%	In vitro	Feng et al. (2020)
						Rice sheath blight	*Rhizoctonia solani*	I:50.9%		
						Anthracnose	*Colletortrichumgloeosporiodes*	I:47.4%		
						Peach shoot blight	*Phomopsis amygdali*	I:51.7%		

EO: EOs; PDI: polydispersity index; O/W NE: oil‐in‐water nanoemulsion; I: % inhibition; PEDC: percentage efficacy of disease control; MIC: minimum inhibitory concentration; IZ: inhibition zone; LC_50_: lethal concentration at the 50%; –: not reported; EC_50_: effective concentration that inhibits 50% of mycelial growth; ED50: estimate the 50% effective dose.

For example, in fennel seeds, Hassanin et al. (2017) demonstrated that reducing thyme EO NE droplet size from ≈207 nm to ≈34.6 nm via extended ultrasonication completely suppressed *S. sclerotiorum* mycelial growth at the lowest tested concentrations. However, the smallest droplets caused germination inhibition at a 1% concentration, indicating a need for dose optimization to avoid phytotoxic effects (Hassanin et al. 2017).

Ali et al. (2017) investigated citronella–neem NE mixtures against *R. solani* and *S. rolfsii*, achieving ED50 values of 25.6 mg/L and 20.9 mg/L, respectively. The mixture outperformed the individual oils, suggesting synergistic effects (Ali et al. 2017).

For black mold and bread mold, Marei et al. (2018) developed citral–chitosan hybrid systems via ultrasonication, producing droplets ranging from 27 to 1283 nm with PDI 0.51–0.61. The most effective formulation (F1) exhibited EC50 values of 278 mg/L against *A. niger* and 221 mg/L against *R. stolonifer* (Marei et al. 2018).

In postharvest protection, de Castro e Silva et al. (2019a) produced neem oil NEs (∼59 nm; PDI ≈0.21) that showed inhibition zones of 28 mm against *A. flavus* and 25 mm against *P. citrinum* without phytotoxicity in soybean seeds (de Castro e Silva et al. 2019a). In a follow‐up study, de Castro e Silva et al. (2019a) incorporated the same NE into pectin‐based seed coatings, improving barrier and mechanical properties while maintaining antifungal activity and seed germination (de Castro e Silva et al. 2019a).

In ornamental crops, Hassanin et al. (2018) reported lemongrass EO NEs that reduced *B. cinerea* severity in poinsettia to 10% (preinoculation) and 13% (postinoculation), compared to 14.5%–18.2% with carbendazim 50% WP. The improved efficacy was attributed to the smaller droplet size and possible activation of plant defense enzymes, including POD and PPO (Hassanin et al. 2018). Although poinsettia is an ornamental plant rather than a food crop, this study was discussed in this work due to its focus on the fungal pathogen B. cinerea, which commonly causes gray mold in a wide range of food crops, including fruits (e.g., berries, grapes, apples, and peaches) and vegetables (e.g., tomatoes, onions, and beans).

D‐limonene, a monoterpene naturally abundant in citrus EOs, has also been investigated as an active ingredient in NE‐based biopesticides. Although many formulations rely on the commercial compound rather than direct extraction from citrus peel oils, its natural origin and recognized bioactivity justify its inclusion as a model “green” pesticide. In rice pathogens, Feng et al. (2020) optimized D‐limonene‐based NEs (10% D‐limonene, 6% EL‐40 emulsifier) by adding the aqueous phase dropwise to the oil–surfactant mixture. Increasing the emulsifier concentration from 2% to 6% improved stability (as indicated by a lower Turbiscan Stability Index) and reduced droplet size, resulting in a formulation that inhibited the growth of *P. oryzae*, *Rhizoctonia solani*, *Colletotrichum gloeosporioides* (Feng et al. 2020), and *Phomopsis amygdali*. The EC50 varied widely by pathogen, ranging from 1.63 µg/mL for *R. solani* to 489 µg/mL for *P. oryzae*. At their respective EC50 values, the NEs consistently outperformed the free oil in terms of inhibition rates.

### 6.2 Herbicidal Activity

Weeds severely reduce crop yield and quality and can act as reservoirs for insects and pathogens (Table 3). Beyond direct competition, synthetic herbicides raise several concerns: residues can persist in soil and contaminate groundwater, leading to accumulation in agricultural products (Jabran et al. 2015); resistance has accelerated in multiple weed species (Travlos et al. 2020); and nontarget injury can occur in rotational crops (Burke and Bell 2014). EOs, rich in natural phytotoxins and allelochemicals, have been reported as effective “natural herbicides” with diverse modes of action and a favorable environmental profile (Dayan and Duke 2014). When nanoencapsulated in natural polymers, EOs can be engineered as target‐specific molecules for receptors in weed root systems, thereby reducing phytotoxicity to adjacent crops (Jampílek and Kráľová 2017). In addition, nanoencapsulation improves EO stability against environmental degradation (e.g., photolysis, volatilization) and can enhance biological activity by preserving phytochemicals and optimizing their release profile (Taban et al. 2020, 2021).

**TABLE 3:** Potential herbicidal activity of green O/W nanoemulsions against weeds in food crops for sustainable management.

Herbicidal activity—Weed control								
Biopesticide			O/W nanoemulsion			Herbicidal activity		
Active ingredient in O/W NE	Plant specie	Main phytochemicals	Mean droplet size	PDI value	Zeta potential	Target weed	Inhibitory effect on seed germination	Refs.
Cultivated thyme EO	*T. capitatus*	Thymol, o‐cymene, α‐terpinene, Trans‐caryophyllene	22.1 nm	—	—	*Convolvulus arvensis*	ED50: 3.465 µg/mL	Balah and Abd El Azim (2016)
Wild thyme EO	*Thymus capitatus*	Thymol, α‐terpinene, 1‐4‐terpineol	12.0 nm	—	—		ED50: 2.616 µg/mL	
Marjoram EO	*Majorana hortensis*	Cis, trans‐sabinene hydrate, α‐terpinene, sabinene	5.3 nm	—	—		ED50: 0.392 µg/mL	
Garden savory EO	*Satureja hortensis*	Carvacrol (55.6%) and γ‐terpinene (31.9%)	< 130 nm even after storage for 30 d.	0.13	—	*Amaranthus retroflexus* ^a^	95%, 800 µL/L	Hazrati et al. (2017)
						*Chenopodium album*	83.4%, 1000 µL/L	
PHCE plus PKOE	*Parthenium hysterophorus*	PHCE: Allelochemical parthenin; PKOE: fatty acid ester	140.10 nm	0.07–0.08 after 60 days	−32.7 to −26.8 after 60 days	*Diodia ocimifolia*	100%, ED50: 5 g/L	Zainuddin et al. (2019)
Savory EO	*Satureja hortensis*	carvacrol (52.55%), γ‐terpinene (30.21%), and α‐terpinene (4.99%)	87 nm	0.210	—	*Amaranthus retroflexus* (as model)	100%, 15 mL/L^b^	Taban et al. (2020)
			70–73 nm, after 6 weeks	0.21–0.28, after 6 weeks	—		100%, 3 mL/L^c^	Taban et al. (2021)
Fennel EO	*Foeniculum vulgare*	Estragole, anethole	< 130 nm	—	—	Weeds of *Triticum aestivum* (wheat)	100%, ED50: 0.05 wt%	Kaur et al. (2021)
Peppermint EO—2%	*Menthae piperitae*	Menthol (44.2%) menthone (27.4%)	100 nm	< 0.1	—	Barnyard grass in maize	ED50: 10% of necrosis in maize and 1.1% in barnyard grass	Rys et al. (2022)

EO: essential oils; PDI: polydispersity index; ED50: herbicide rate required to cause 50% reduction in seed germination; PHCE: *Parthenium hysterophorus* L. crude extract; PKOE: palm kernel oil ester; ^a^: under greenhouse conditions; ^b^: EO nanoencapsulated using Persian gum cross‐linked by citric acid; ^c^: EO nanoencapsulated using Arabic gum‐gelatin and apple pectin biopolymers cross‐linked by citric acid.

Macroemulsions and NEs from *Thymus capitatus* (wild and cultivated) and *Majorana hortensis* EOs were prepared and tested to control *Convolvulus arvensis* and *Setaria viridis*. NEs prepared with Tween 20/Tween 80 and chloroform as cosurfactant exhibited droplet sizes of 5.3–22.4 nm and high stability for over 5 months. Postemergence assays showed that marjoram EO NEs provided the most potent inhibition of *C. arvensis* growth under greenhouse conditions, highlighting the importance of oil type, formulation, and application stage (Balah and Abd El Azim 2016).

*Satureja hortensis* EO NEs were investigated for controlling *Chenopodium album* and *Amaranthus retroflexus*. Using Persian gum as a wall material cross‐linked with citric acid, the NEs achieved potent inhibition of seed germination and seedling growth, attributed to oxygenated monoterpenes like carvacrol and thymol (Hazrati et al. 2017).

Building on this, Taban et al. (2020) encapsulated *S. hortensis* EO in Persian gum‐based nanocapsules and showed herbicidal activity against tomato (*Lycopersicon esculentum* Mill.) and amaranth (*Amaranthus retroflexus* L.), achieving 100% *A. retroflexus* control at 15 mL/L within 48 h. Later, Taban et al. (2021) optimized the encapsulation using Arabic gum–gelatin and apple pectin, improving efficiency (72.1%–92.8%), stability (70–73 nm; PDI 0.21–0.28), and reducing the EO concentration needed for complete control to 3 mL/L (Taban et al. 2020, 2021).

Zainuddin et al. (2019) designed a palm kernel oil ester (PKOE)‐based NE containing *Parthenium hysterophorus* crude extract for pre‐emergence control of *Diodia ocimifolia*. A key finding from this work was the demonstration that vegetable oils, specifically PKOE, can act as multipurpose agents—serving both as a natural solvent and as an herbicide adjuvant—while enhancing their activity through cell membrane disruption. The NE inhibited germination at 5 g/L, compared to 10 g/L for the crude extract (Zainuddin et al. 2019). Additionally, the fatty acid ester from PKOE can enhance the herbicidal activity, acting similarly to petroleum oils by disrupting the cell membranes of weed plants in their surface cells, resulting in the death of plant tissue (Vaughn and Holser 2007).

*Foeniculum vulgare* EO components—estragole, anethole, and their mixture—encapsulated in NE showed higher effectiveness and completely inhibited seed germination of several weeds of *Triticum aestivum* (wheat): *Phalaris minor, Avena ludoviciana, Rumex dentatus*, and *Medicago denticulat*a even at 0.05 wt%, likely due to membrane leakage and reactive oxygen species‐mediated cellular damage (Kaur et al. 2021).

Barnyard grass is a persistent invasive weed that affects crop yield and quality, especially in maize (*Zea mays*) cultivation. A peppermint EO‐based NE stabilized with Eco‐Polysorbate 80 demonstrated selective herbicidal activity, effectively suppressing barnyard grass while causing minimal damage to maize at a 2% concentration, highlighting its potential for sustainable weed management in food crops (Rys et al. 2022).

### 6.3 Acaricidal Activity

Phytophagous mites, such as Tetranychus urticae, Aceria oleae, and Tyrophagus putrescentiae, are significant agricultural pests that cause extensive damage to crops and stored products. Conventional acaricides often rely on synthetic chemicals with environmental and health concerns, making plant‐derived NEs an attractive alternative to conventional pesticides in the management of mites. Some studies have explored O/W NEs incorporating EOs or isolated monoterpenes as active agents, emphasizing the absence of toxic organic solvents and the use of biocompatible surfactants (Table 4).

**TABLE 4:** Potential acaricidal activity of green O/W nanoemulsions against spider mites in food crops for sustainable management.

Acaricidal activity against spider mites										
Biopesticide			O/W NE properties			Acaricidal effect				Refs.
Active ingredient	Plant species derived	Phytochemicals	Mean droplet size	PDI value	Zeta potential	Plant host; symptoms and damages	Target mite	LC_50_ values	Food crop tested	
Garlic EO—5%	*Allium sativum*	Organosulfur compounds	93.4 nm	—	−31.67 ± 2.4 mV	Host: olive; Irregular coloring/deformation of leaves and fruits	*Aceria oleae*	298.225 µg/mL^a^	Olive leaves	Mossa et al. (2018)
							*Tegolophus hassani*	309.634 µg/mL^a^		
Bottlebrush EO—10%	*Callistemom viminalis*	Eucalyptol, α‐pinene	10 nm	0.510	—	Host: food crops; Wilting, tissue death, leaf deformity, and abscission disrupted photosynthesis.	*Tetranychus urticae*	5000 mg/L^b,c^	Bean plants (*Phaseolus vulgaris*)	Badawy et al. (2018)
Oregano EO—10%	*Origanum vulgare*	Pulegone, menthone	8.9 nm	0.249	—					
Pulegone (compound)—10%	—	Pulegone 97%	7.0 nm	0.620	—					
Limonene (compound)—10%	—	R‐limonene 85%	8.10 nm	0.447	—					
Basil EO—10%	*Ocimum basilicum*	Methyl eugenol	78.5 nm	0.18	—	Host: stored grains; in Soybean plants: Root staining, few roots, leaf mosaic, wilting, and death.	*Tyrophagus putrescentiae*	2.2 µL/cm^2 d,e^	—	Al‐Assiuty et al. (2019)
Achillea EO—10%	*Achillea fragrantissima*	Cis, trans‐Thujone; Artemisia ketone; santolina alcohol	91.3 nm	0.20	—			4.7 µL/cm^2 d,e^		
Achillea EO—10%	*Achillea santolina*	Fragranyl acetate; 1,6‐Dimethyl‐1,5‐cyclooctadiene; 1,8‐cineole	104.6 nm	0.26	—			9.6 µL/cm^2 d,e^		

EO: essential oil; PDI: polydispersity index; ^a^: 48‐h LC_50_ values; ^b^: 100% reduction at 5000 mg/L after 2–3 days of application on bean plants (*Phaseolus vulgaris* L.); ^c^: under greenhouse conditions ^d^: 48‐h LC_50_ values; ^e^: LC_50_ for benzyl benzoate, a standard acaricide was 9.8 µL/cm^2^.

O/W NEs (10% oil phase) containing *Callistemon viminalis* EO, *Origanum vulgare* EO, R‐limonene, or pulegone, were prepared for controlling two‐spotted spider mite (*T. urticae*) on bean plants. The NEs, produced via high‐energy ultrasonication with polysorbate surfactants, exhibited tiny droplet sizes (7.07–10.18 nm) and PDI values between 0.249 and 0.620. All formulations demonstrated high acaricidal activity, with efficacy varying according to the active compound; *O. vulgare* EO and pulegone were the most potent (Badawy et al. 2018).

Garlic (*Allium sativum*) EO NEs (5% oil phase) stabilized by polysorbates were developed to target two eriophyid olive mites—*A. oleae* and *Tegolophus hassani*. The formulations were stable and rich in organosulfur compounds, particularly diallyl disulfide and diallyl trisulfide, which were associated with strong acaricidal effects. Preliminary in vivo assays suggested low mammalian toxicity, supporting the safety profile of garlic EO‐based nanoacaricides (Mossa et al. 2018).

O/W NEs containing 10% *Ocimum basilicum* EO were evaluated against the mold mite *T. putrescentiae*. Using high‐energy emulsification, they obtained stable NEs that achieved an LC_50_ of 2.2 µL/cm^2^ air in fumigation assays, which is lower than the standard acaricide benzoyl benzoate (LC_50_ of 9.8 µL/cm^2^). The high activity was attributed to oxygenated monoterpenes such as linalool and eugenol (Al‐Assiuty et al. 2019).

### 6.4 Repellency and Insecticidal Effect

Insecticides comprise a broad class of pesticides designed to kill, repel, or disrupt the life cycle of insect pests. This section addresses both insecticidal and repellent effects of nanoemulsified phytochemicals, highlighting their mechanisms of action, efficacy, and agricultural relevance. Several studies are focused on investigating the repellency effect against insects (Cantó‐Tejero et al. 2021; Giunti et al. 2019; Lima et al. 2021; Nogueira et al. 2020; Sakulku et al. 2009) and aphids (Pascual‐Villalobos et al. 2017), beyond the insecticidal (ovicidal, larvicidal, adulticidal) activity of these nanobiopesticides. Despite the properties of plant volatiles as insect repellents, there is a lack of such products for the organic sector (Pascual‐Villalobos et al. 2017). This section emphasizes the pesticidal applications of green O/W NEs at the crop production stage, given their critical role in ensuring food security and minimizing chemical residues at the origin of the food chain. Thus, these eco‐friendly strategies can significantly reduce the need for conventional pesticides, improve preharvest safety, and align with sustainable food production systems.

#### 6.4.1 Aphicidal Activity

Aphids (*Aphididae*) represent a major agricultural pest group due to their direct feeding damage, honeydew secretion, and ability to transmit numerous plant viruses. Conventional control relies heavily on synthetic insecticides; however, their intensive use has led to issues such as the development of resistance, environmental contamination, and adverse effects on nontarget organisms. The organic agriculture sector requires alternative aphicides that prevent or repel these pests. In this context, EOs rich in terpenoids and phenolic compounds have emerged as promising aphicidal agents, and their formulation as NEs can enhance dispersion, penetration, and overall bioefficacy.

A lemon oil‐based NE system was developed as a carrier for natural pyrethrins derived from Tanacetum cinerariifolium, using polysorbates, glycerol, and water to generate kinetically stable O/W droplets upon dilution. Structural analysis confirmed the formation of two droplet populations (approximately equal to 36 nm and greater than 150 nm), with evidence that the biopesticide was incorporated into the interfacial nanostructure. When tested against Aphis gossypii on eggplant, the pyrethrin‐loaded NEs exhibited higher insecticidal efficacy than commercial formulations, indicating that nanoencapsulation enhanced the bioavailability and field performance of the botanical insecticide (Kalaitzaki et al. 2015).

The bird cherry‐oat aphid, *Rhopalosiphum padi* L., is probably the major pest of temperate cereal crops on a world scale, as it attacks all the major cereals and pasture grasses. Pascual‐Villalobos et al. (2017) prepared low‐energy O/W NEs from a set of EOs and selected pure EO constituents to test them against the bird cherry–oat aphid, *R. padi* L. Laboratory bioassays showed dose‐dependent aphid control with both mortality and repellency components. Nanoemulsifying carvone increased mobility, while cis‐jasmone repelled *R. pad*i at a very low dose (0.02 µL/cm^2^ of the treated leaf). Performance depended strongly on chemical composition: some pure compounds matched or exceeded the activity of their parent oils, while specific EO blends exhibited additive effects. The study highlights composition‐driven efficacy and supports nanoemulsification as a means to enhance biological performance without relying on toxic solvents (Pascual‐Villalobos et al. 2017).

In this sense, O/W NEs of EO‐derived actives—namely, (E)‐anethole, farnesol, and (Z)‐jasmone—were formulated using Tween 80 and evaluated their performance against *Myzus persicae* and *Macrosiphum euphorbiae* (RD_50_ = 0.011–0.086 µL/cm^2^). Two‐choice olfactometer assays and on‐plant tests demonstrated significant repellency at concentrations of 0.1%–0.3% (v/v) and reductions in instantaneous population growth. Contact bioassays confirmed activity, while residual toxicity on beneficials (e.g., *Aphidius colemani* adults and *Sphaerophoria rueppellii* larvae) was low at the tested doses, supporting compatibility with IPM programs (Cantó‐Tejero et al. 2021).

Abdelaal et al. (2021) provided a more detailed mechanistic evaluation using four EO NEs (basil, cumin, marjoram, and chamomile) against *Aphis craccivora*. NEs dramatically reduced the LC_50_ values relative to the crude oils (e.g., basil oil LC_50_ = 992 mg/L vs. basil NE LC_50_ = 45 mg/L), approaching the efficacy of synthetic comparators such as dinotefuran (LC_50_ = 4.6 mg/L). Importantly, biochemical assays revealed enzyme inhibition patterns in aphids, including suppression of acetylcholinesterase and β‐esterases, alongside alterations in detoxification enzymes (GST and MFO), suggesting multiple modes of action (Abdelaal et al. 2021). These metabolic disruptions may underlie the higher potency and lower resistance risk associated with nano‐formulated EOs.

Stable NEs using spearmint EO (Mentha spicata) and its principal constituent, carvone (81.88%), to control aphid pests (Rhopalosiphum maidis and Sitobion avenae) were developed (Mondal et al. 2024). The formulations were prepared via low‐energy spontaneous emulsification using Triton X‐100, yielding NEs with average droplet sizes of 22.1 nm (EO) and 41.2 nm (carvone), and PDI values of 0.51 and 0.53, respectively. Despite their relatively low zeta potential (−0.33 mV for EO and −9.98 mV for carvone), the NEs exhibited high physical stability attributed to steric stabilization. Although both formulations demonstrated significant aphidicidal and repellent activity, the carvone‐based NE showed greater efficacy (LC_50_ as low as 0.87 mg/mL).

**FIGURE 4:**
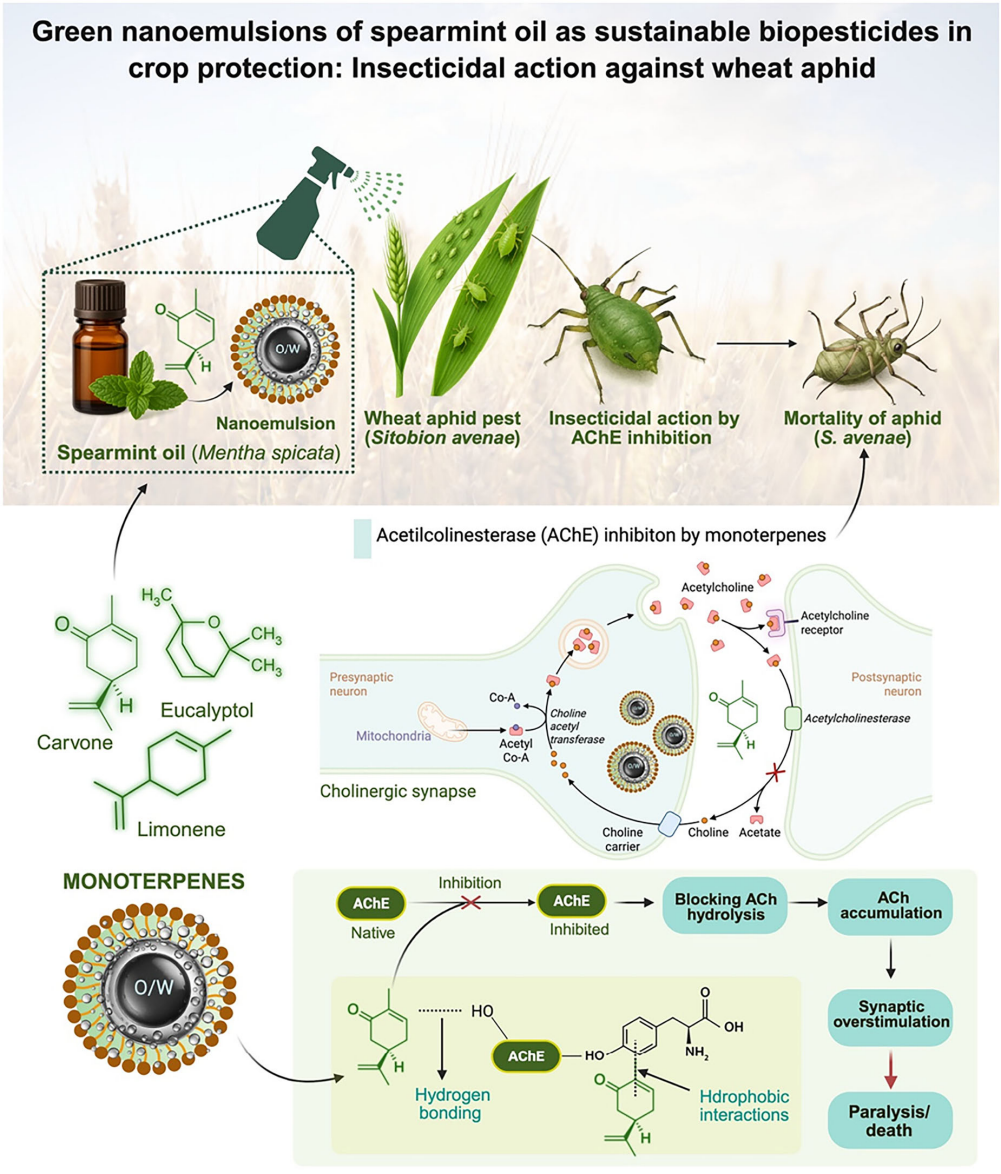
Proposed mechanism of insecticidal action of spearmint oil (*Mentha spicata*) and carvone nanoemulsions against wheat aphid (Sitobion avenae). Carvone interacts with the acetylcholinesterase (AChE) active site through hydrogen bonding and hydrophobic interactions, thereby blocking the hydrolysis of acetylcholine (ACh). This inhibition leads to ACh accumulation, synaptic overstimulation, and aphid paralysis/death, supporting the potential of green nanoemulsions as sustainable biopesticides in crop protection.

Additionally, in vitro acetylcholinesterase inhibition assays and molecular docking studies confirmed that carvone and other monoterpenes (e.g., limonene, eucalyptol) interact effectively with the AChE active site through hydrophobic interactions and hydrogen bonding, supporting the mechanism of insecticidal action that leads to synaptic overstimulation and insect mortality (Mondal et al. 2024). Figure 4 summarizes the proposed mechanism, integrating NE formulation, phytochemical interactions with AChE, and the resulting control of the wheat aphid (Sitobion avenae). The neuronal scheme in the figure is a simplified representation of a cholinergic synapse, intended to illustrate the general mechanism of AChE inhibition in insects.

Most recently, the potential of NEs containing purslane seed oil (Portulaca oleracea), radish seed oil (Raphanus sativus), and rosemary EO (Rosmarinus officinalis) was evaluated for controlling major aphid pests (Abd‐Elnabi et al. 2025). Purslane oil was the most effective, chemically characterized by aromatic hydrocarbons, including 1‐methyldecyl‐ and 1‐methyldodecyl‐benzene derivatives. The formulations remained physically stable under stress conditions (freeze–thaw, centrifugation, heating–cooling), without phase separation, sedimentation, or creaming, and exhibited improved pesticidal activity compared to bulk oils, especially against A. gossypii (Abd‐Elnabi et al. 2025).

Taken together, these studies reinforce that NEs of plant‐derived EOs enhance the aphicidal spectrum across different species (*M. persicae*, *A. gossypii*, and *A. craccivora*), acting through both physical delivery advantages and biochemical interference. This dual action positions them as promising eco‐friendly alternatives for IPM in crops where aphids are key pests.

#### 6.4.2 Insecticidal Activity Against Food Crop Pests

Green O/W NEs have also been explored for crop fields and stored grain pests. In line with sustainable food production goals, this review highlights the insecticidal properties of green O/W NEs at the crop production stage, where their use can directly reduce pesticide residues and enhance food safety from the source.

A consistent body of evidence highlights that Nes carrying EOs and plant extracts can act as effective bioinsecticides in crop protection, particularly by overcoming limitations of volatility, solubility, and instability of natural compounds.

*Planococcus minor*, commonly known as the mealybug related to virus‐infected plants. It is a polyphagous pest affecting estate crops and causing significant crop losses to various food‐producing plants such as citrus, grapes, and coffee. This pest can suck plant sap, weakening plants and transmitting viruses that reduce crop yields. A NE based on *kemiri sunan* (*Aleurites trisperm*a) seed oil demonstrated strong insecticidal activity against *P. minor*, causing up to 90% mortality 5 days after application (Prabowo and Damaiyani 2019). The formulation achieved an LC_50_ of 0.09% and LT_50_ of 3.7 days, indicating potent and relatively fast action. Its low toxicity to nontarget organisms and suitability for integration into pest management programs highlight its potential as a botanical alternative to conventional insecticides.

Crithmum maritimum EO, rich in dillapiole, myristicin, γ‐terpinene, and thymol methyl ether, was also evaluated in NE (O/W) form for its activity against the agricultural pest Spodoptera litura (Suresh et al. 2020). Both the free EO and its nanoencapsulated formulations demonstrated significant larvicidal toxicity, with nanoemulsification enhancing efficacy and reducing developmental fitness parameters such as adult emergence and fecundity. These results highlight the potential of C. maritimum NEs as botanical insecticides against economically relevant lepidopteran pests in crop systems.

Benelli et al. (2020) produced a stable NE (droplet size ∼140 nm, SOR 0.6) encapsulating *Carlina acaulis* root EO, dominated by carlina oxide (> 90%). Both the free EO and the NE displayed strong larvicidal effects against *Lobesia botrana*, the European grapevine moth, with LC_50_ values of 7.29 µL/mL and 9.04 µL/mL, respectively, marking the first report of EO‐based NEs against this economically relevant pest.

The pistachio psylla (Agonoscena pistaciae Burkhardt and Lauterer) is a significant pest in pistachio orchards of Iran. In this study, an O/W NE formulated with methanolic spinach (Spinacia oleracea) seed extract showed markedly higher toxicity against nymphs compared to its nonformulated counterpart, with LC_50_ values dropping from 4381 mg/L to 468 mg/L for early instars, and from 3946 mg/L to 124 mg/L for fifth instars (Mahdavian et al. 2021). Arugula (Eruca sativa) seed oil and a commercial product (Dayabon) were also tested, showing moderate activity. Field assays revealed that botanical treatments remained effective for up to 21 days after application, underscoring their potential as eco‐friendly alternatives to synthetic pesticides in managing pistachio pests.

Anaphothrips obscurus is a globally distributed pest that feeds on cereal crops and grasses, posing a significant threat to agricultural productivity. In a recent study, a binary EO‐based microemulsion (ME) combining methyl salicylate and carvacrol (5:5, v/v) exhibited synergistic fumigant toxicity against both second‐instar nymphs and adults of A. obscurus, with a cotoxicity coefficient of 151.15. The optimized formulation (10% active ingredients) formed a stable, transparent single‐phase system. Field trials demonstrated high control efficacy, reaching 89.2% in pepper and 82.6% in broad bean crops 7 days postapplication at a dose of 600 g a.i./ha, supporting its potential as an effective botanical alternative to synthetic insecticides for thrips management.(Lu et al. 2020).

A neem oil‐based O/W NE was optimized using natural adjuvants (*Cymbopogon citratus* and *Prosopis juliflora*) to overcome stability limitations commonly associated with botanical pesticides (Iqbal et al. 2021). The formulation containing 30% *P. juliflora* extract exhibited the most favorable physicochemical profile, with droplet sizes of 25–50 nm, zeta potential around –30 mV, PDI < 0.3, and high kinetic stability without phase separation. FTIR confirmed the integrity and compatibility of azadirachtin within the NE matrix, while degradation remained minimal (1.42%), with an extended half‐life of nearly 493 days. In in vivo assays on brinjal (*Solanum melongena*), the optimized NE achieved 91.2% control of whitefly (*Bemisia tabaci*), demonstrating that natural adjuvants can enhance both the stability and field efficacy of neem‐based biopesticides for integration into IPM strategies.

Other field investigations confirmed the broad potential of NEs formulations against crop pests. Field trials conducted in two agricultural regions of Côte d'Ivoire (Yamoussoukro and Korhogo) demonstrated the effectiveness of a NE formulated with Lippia multiflora EO in managing multiple cabbage pests, including Plutella xylostella, Brevicoryne brassicae, Hellula undalis, Spodoptera exigua, and Bemisia tabaci (Tia et al. 2023). When applied under wet‐season field conditions, the NE significantly reduced pest populations compared to untreated plots and achieved control levels comparable to those of the synthetic insecticide lambda‐cyhalothrin (Karate 5 EC). The most notable reductions were observed for B. brassicae and P. xylostella, where pest densities decreased to near‐zero levels in plots treated with NE. Yield improvements and reduced head damage further confirmed the agronomic relevance of the treatment, indicating that L. multiflora‐based NEs represent a promising botanical alternative for integration into sustainable pest management programs for *Brassica* crops (Tia et al. 2023).

The pesticidal effects are likely attributed to the rich content of monoterpenes in L. multiflora oil, which may act through multiple mechanisms such as inhibition of acetylcholinesterase activity (Mondal et al. 2024), disruption of insect neurotransmission, interference with cuticle permeability, or feeding deterrence—effects potentially enhanced by nanoencapsulation through increased solubility and bioavailability. A previous study published by Fernandes et al. (2014) also developed a NE containing the apolar fraction of *Manilkara subsericea* fruits (rich in triterpenes), which induced significant mortality in the cotton stainer bug *Dysdercus peruvianus*, a major pest of cotton crops, while showing no acetylcholinesterase inhibition and no mammalian toxicity.

Giuliano et al. (2024) developed a garlic EO‐based NE for controlling Spodoptera littoralis larvae, a major lepidopteran pest in horticulture. The NE allowed reduced doses and prolonged field activity, although some phytotoxicity was observed in treated plants. It was formulated with a high EO content (15%) and a low concentration of surfactant (Tween 80, 5%), using magnetic stirring and ultrasonication techniques to achieve physical stability and homogeneity. The resulting formulation exhibited a mean droplet size of 141.0 ± 1.4 nm, low PDI (0.146 ± 0.009), and a zeta potential of −27.4 ± 1.9 mV, indicating a stable and uniform dispersion. The formulation demonstrated significant larvicidal and antifeedant effects at low concentrations, with LC_50_ and LC_90_ values of 1.72% and 2.79%, respectively, after 24 h (Giuliano et al. 2024).

These studies demonstrate that O/W NEs offer a robust and scalable approach for eco‐friendly pest management. By improving the solubility, stability, and targeted delivery of lipophilic phytochemicals, these systems bridge the gap between efficacy and environmental safety. In several cases, they matched or even surpassed the performance of synthetic pesticides—often at reduced doses, with extended residual activity, and lower toxicity to beneficial organisms. Their relevance extends not only to sustainable agriculture but also to the future of IPM programs.

## 7 Toxicological Concerns of O/W Nanoemulsions in Food Safety

While O/W NEs formulated with EOs and plant extracts are gaining attraction as sustainable alternatives to conventional pesticides, toxicological assessments remain limited, particularly in relation to food safety.

Recent toxicological evaluations have demonstrated that nanoencapsulation can mitigate key safety concerns associated with the use of conventional pesticides. For example, Mancozeb—a broad‐spectrum fungicide known for its high toxicity—was nanoformulated with eugenol, and the resulting NEs were assessed for cytotoxicity, genotoxicity, and ecotoxicity using healthy human cells and springtail bioassays (da Silva Gündel et al. 2019). While the free compounds exhibited significant toxicity, especially to soil organisms and human cells, their nanoencapsulated forms showed reduced adverse effects, likely due to controlled release and decreased environmental mobility. A garlic EO‐based NE developed against A. gossypii demonstrated high insecticidal efficacy with low lethal doses, while showing negligible phytotoxicity in pepper plants. However, significant toxicity to honeybees was observed via ingestion (Modafferi et al. 2025). These findings highlight the potential of NEs to reduce pesticide residues in food and minimize unintended impacts on nontarget organisms, contributing to safer pest control strategies in sustainable agriculture.

However, most studies focus on efficacy against crop pests but rarely evaluate the potential for phytochemical residues in edible plant tissues, bioaccumulation, or long‐term dietary exposure risks. This gap is especially relevant given the increased solubility and bioavailability conferred by nanoencapsulation, which may influence absorption, metabolism, and toxicity profiles of the active compounds. Moreover, the interactions between nanoemulsified biopesticides and food matrices, as well as the effects of processing conditions and storage stability, are poorly understood. To advance the adoption of NE‐based biopesticides in sustainable food production systems, future research should incorporate food‐grade safety standards, residue quantification, and comprehensive toxicological profiling, ensuring alignment with consumer protection regulations and public health goals.

## 8 Trends, Challenges, and Knowledge Gaps

Formulation strategies strongly influence the performance of biopesticidal NEs. For example, premixing the emulsifier into the oil phase and adding water dropwise improved NE stability in D‐limonene systems (Feng et al. 2020). Additionally, increasing the emulsifier concentration also reduced droplet size and prevented phase separation. The droplet size–efficacy relationship is evident in several studies: thyme EO NEs at ∼34.6 nm were more effective than ∼207 nm (Hassanin et al. 2017), although smaller droplets may increase phytotoxic risk. Sensitivity varies considerably between fungal species, as seen in the extensive EC50 range for D‐limonene NEs.

Although droplet size reduction is often associated with enhanced bioavailability and biological performance, several studies indicate that nanoemulsification does not universally translate into increased efficacy. In some cases, comparable pesticidal outcomes were observed across formulations with different droplet sizes, suggesting that oil composition, active compound chemistry, target organism, and application mode may play equally or more decisive roles than droplet size alone (Feng et al. 2020; Hassanin et al. 2017). This variability highlights that droplet size is not a standalone predictor of efficacy, and its impact must be interpreted within the broader context of formulation and biology.

Postharvest and seed protection applications are particularly promising, as neem NEs function both as direct antifungals and as active ingredients in edible coatings, delivering dual benefits for disease control and seed quality preservation (de Castro e Silva et al. 2019a).

The studies related to phytopathogenic bacteria collectively demonstrate that NE formulation enhances the antibacterial activity of monoterpenes and can induce host resistance responses, offering both direct and indirect protection. However, the phytotoxicity observed with carvacrol NEs underscores the need to establish safe application rates. Moreover, hybrid systems incorporating biopolymers, such as chitosan, may provide synergistic effects by combining physical disruption of bacteria with biochemical inhibition. Finally, comparative analyses across multiple monoterpenes suggest that compound‐specific physicochemical properties dictate NE performance and should guide formulation design.

The reviewed studies also highlight the versatility of EO‐based NEs for weed management, with effects influenced by oil composition, formulation stability, and application method.

One of the main challenges in advancing green NE technology is the continued reliance on conventional synthetic surfactants, such as alkylphenol ethoxylates (APEOs; e.g., Triton X‐100), polysorbates (e.g., Tweens), and sorbitan fatty acid esters (e.g., Spans). Although these are widely accepted in food and agrochemical formulations due to their emulsifying efficiency and regulatory approval, they are derived from petrochemicals and do not meet the criteria of green chemistry. Moreover, their environmental behavior is not fully understood. While they may enhance foliar uptake, studies show that their presence can unpredictably affect pesticide solubility, volatilization, and mobility in soil (Katagi 2008). Their impact on hydrolysis, photolysis, and root uptake in real‐world agricultural systems remains poorly studied, and their role in bioconcentration and trophic transfer in aquatic environments is a growing concern (Katagi 2008). These knowledge gaps reinforce the need to develop and validate biodegradable, bio‐based surfactants that ensure both efficacy and environmental safety in sustainable crop protection strategies. Encapsulation techniques using natural or bio‐based surfactants (e.g., saponins, gums, pectins, and lecithins) are promising for organic systems, as they reduce the required doses and prolong shelf life. However, variability in weed susceptibility, potential crop phytotoxicity, and scalability of production remain key challenges for field adoption.

Regarding biopesticide NEs specifically for controlling mites and ticks, studies reviewed indicate that NE formulations enhance acaricidal potency by improving the solubility and dispersion of EO components (Al‐Assiuty et al. 2019). The extremely small droplet sizes (< 10 nm) reported by Badawy et al. (2018) suggest potential for deep penetration into mite cuticles. However, field‐scale validation is still lacking, and compound‐specific optimization is required to balance efficacy, stability, and safety for nontarget organisms.

The EO/phytochemical choice and NE composition largely determine aphid outcomes (mortality vs. repellency and their magnitude). Low‐energy NE methods with food‐grade surfactants are promising for scalability and regulatory acceptance. Remaining needs include field validation across aphid species, dose optimization to balance efficacy and crop safety, and cost‐effective production routes.

Despite promising advances, most O/W NE formulations still rely on synthetic surfactants, which limits their classification as truly “green” technologies. Only a minority of studies employ natural or food‐grade emulsifiers, such as saponins, lecithins, or polysaccharides, indicating a need for a deeper exploration of biocompatible formulation systems. Another significant gap lies in the lack of studies targeting critical pests, such as nematodes and plant viruses, which remain underexplored in NE research. Furthermore, challenges persist in scaling up production, maintaining physical and chemical stability under field conditions, and ensuring regulatory acceptance. Future research should prioritize cost‐effective, biodegradable formulations with validated long‐term field performance, especially in diverse climates and cropping systems.

It is worth noting that the majority of studies included in this review originate from Brazil, the United States, and Europe, reflecting the current distribution of indexed research on NE‐based biopesticides. In contrast, Asia and Africa remain underrepresented, despite their critical importance for sustainable crop protection. This geographic imbalance highlights a significant knowledge gap and underscores the need for increased research investment, region‐specific field validation, and formulation strategies adapted to local crops, climatic conditions, and regulatory frameworks.

## 9 Food Safety and Sustainability Perspective

Green O/W NEs represent a promising frontier in the delivery of phytochemical‐based biopesticides for sustainable crop management. By enhancing the solubility, stability, and bioefficacy of natural compounds—such as EOs and plant‐derived actives—these systems offer eco‐compatible solutions that reduce the need for synthetic pesticides. This shift supports not only crop protection but also the broader goals of food safety and environmental sustainability.

From a food systems perspective, the use of bio‐based nanoformulations can contribute to lowering pesticide residues in food products, minimizing contamination risks to nontarget organisms, and aligning with organic and low‐input farming practices. However, the widespread adoption of these technologies still requires careful attention to toxicological safety, regulatory frameworks, and standardization of formulation strategies to ensure scalability and consumer trust.

Moreover, advancing green NEs as tools for IPM aligns with the SDGs, particularly those targeting zero hunger, responsible production, and environmental protection. Future innovation should prioritize fully biodegradable ingredients, robust ecotoxicological assessments, and multifunctional delivery systems that integrate seamlessly into sustainable food production chains.

In this context, green NEs hold strategic value not only for enhancing food crop yields but also for promoting safer, more resilient, and environmentally sound agri‐food systems.

## 10 Commercial Outlook and Regulatory Challenges

Although still largely in the academic or precommercial stage, a few biopesticidal products incorporating O/W NEs have begun to emerge, especially those based on EOs such as citronella and neem. These products often claim enhanced efficacy and lower residue levels; however, few disclose detailed formulation data due to proprietary constraints.

Most green O/W NEs remain at the academic or pilot scale, and a limited number of commercial biopesticides based on neem or EOs are already available. Neem‐based products, such as refined azadirachtin formulations (e.g., NeemAzal), are marketed as O/W emulsions exhibiting submicron droplet sizes, which enhance stability, bioavailability, and field performance, even when not explicitly labeled as NEs (Trifolio‐M GmbH 2015). Moreover, while it is primarily registered in Europe as an insecticide and acaricide, multiple studies have reported its potential to suppress plant pathogens, with antifungal activity being associated with the presence of fungitoxic phenolic constituents (Oliveira et al. 2025).

Similarly, citronella‐based biopesticides and repellents are commercially formulated as O/W emulsions using nonionic surfactants, with droplet dimensions reported in the submicron or nanometric range (Citrefine International Ltd 2025; Mosi‐guard 2025). In many cases, the absence of explicit “nano” labeling reflects regulatory and marketing considerations rather than fundamental differences in formulation technology.

Commercial citronella‐ and plant‐based repellents, such as Mosiguard Lemon Eucalyptus (Citriodiol‐based), as well as other EO‐based products, are currently available on the market(Citrefine International Ltd 2025; Mosi‐guard 2025). These formulations are often designed to improve efficacy and persistence through advanced emulsification strategies, including microemulsion‐like systems, even when not explicitly labeled as such. Citriodiol is a commercially available, plant‐based insect repellent derived from the oil of the *Eucalyptus citriodora* tree and enriched in p‐menthane‐3,8‐diol (PMD), a naturally occurring compound responsible for its repellency effect. Notably, Citriodiol‐based products have demonstrated repellency performance comparable to synthetic repellents such as DEET under specific conditions, highlighting the translational potential of optimized oil‐based delivery systems (Citrefine International Ltd 2025; Misni et al. 2017).

It is important to note that, despite the use of natural active ingredients and food‐grade formulation components, most commercial neem‐ and EO‐based biopesticides rely on conventional synthetic emulsifiers and surfactants. As such, these products can be considered “green” primarily in terms of reduced toxicity and renewable actives, but they do not fully comply with strict green chemistry principles regarding formulation composition. This distinction highlights a critical gap between currently available commercial products and the fully bio‐based green O/W NE systems emphasized in this review.

Economic feasibility remains a critical consideration. While NEs can reduce the required doses of active compounds and prolong efficacy, the cost of nanoformulation (including equipment, stabilizers, and regulatory compliance) may offset these benefits unless optimized at scale.

From a regulatory perspective, the commercialization of green NE‐based biopesticides remains challenging, primarily due to their positioning at the intersection of nanotechnology and botanical pest control. These systems are typically designed to deliver EOs and plant‐derived extracts—complex, multicomponent active substances with recognized pesticidal potential—using nanoscale O/W structures that can significantly alter exposure profiles, bioavailability, and environmental fate.

In the EU, plant protection products are regulated under Regulation (EC) No. 1107/2009 (European Parliament and Council of the European Union 2009), which may trigger nano‐specific evaluation requirements when engineered nanomaterials are involved (European Commission 2018; Kah et al. 2013). Regulatory expectations have also been shaped by guidance from the European Food Safety Authority (EFSA) on the application of nanoscience and nanotechnologies, which outlines extensive physicochemical and toxicological data requirements for risk assessment (European Economic and Social Committee—EESC 2011). However, such guidance was primarily developed for conventional nanomaterials and single‐molecule actives, and its application to dynamic, soft nanostructures delivering complex botanical mixtures—such as EOs and plant extracts—poses practical challenges related to characterization, standardization, and cost‐effective compliance.

In the United States, the Environmental Protection Agency (EPA) currently evaluates nano‐enabled pesticide formulations under existing pesticide legislation on a case‐by‐case basis, focusing on potential changes in exposure and environmental fate associated with nano‐enabled delivery systems (Ranjani et al. 2025; Science Policy Council 2017; U.S. Environmental Protection Agency 2011; U.S. Food and Drug Administration 2014), rather than on the botanical origin or “greenness” of the active ingredients themselves. As a result, green NEs based on EOs and plant extracts are assessed using regulatory logics developed for conventional or nano‐enabled pesticides, without specific provisions addressing the unique variability and compositional complexity of botanical actives.

In many countries outside the EU and the United States, specific regulatory frameworks for nanotechnology‐based plant protection products are still lacking. In these contexts, NE‐based biopesticides containing EOs or plant extracts are generally evaluated under conventional pesticide regulations, without dedicated guidance for nanoscale characterization or for the combined assessment of nano‐enabled delivery systems and complex botanical actives. This regulatory gap has been widely recognized in international assessments. It underscores the need for harmonized regulatory approaches that explicitly consider the dual nature of green NEs—as both nano‐enabled systems and carriers of plant‐derived bioactive mixtures—to support their safe and responsible translation from laboratory research to field applications (Cardoso e Bufalo et al. 2025; FAO and WHO 2011, 2013; Kah et al. 2013; OECD—Organisation for Economic Co‐operation and Development 2025; Ranjani et al. 2025).

## 11 Conclusion and Outlook

Natural NEs have already shown validated efficacy in agriculture through a wide range of pesticidal activities, such as antimicrobial, aphicidal, herbicidal, fungicidal, acaricidal, and insecticidal effects, using EOs, plant extracts, and other phytochemicals. Their proven advantages include increased solubility of hydrophobic actives, enhanced bioavailability, controlled release, and long‐term physical stability. Specific targets, such as *Aphis gossypii*, *Spodoptera littoralis*, and *Fusarium oxysporum*, have been successfully managed in greenhouse and in vitro studies. However, these advantages must be carefully balanced against well‐documented challenges, including phytotoxic effects associated with highly bioavailable nanoemulsified actives, formulation‐dependent crop sensitivity, and the need to establish safe application thresholds under realistic field conditions.

No studies targeting phytopathogenic viruses or nematodes were identified in this review, underscoring a critical research gap in the application of green NEs for the integration of pest management.

Stable O/W NEs, both based on nonbiodegradable food‐grade surfactants (e.g., polysorbates) or biodegradable ones (e.g., saponins), have demonstrated effectiveness even under stress conditions, such as heating–cooling cycles and freeze–thaw storage. Nevertheless, despite the conceptual focus on green and bio‐based NEs, a substantial number of studies still rely on conventional surfactants—particularly Tween and Span—as emulsifying agents. This reliance highlights a disconnect between the intended sustainability goals and current formulation practices, underscoring the need for further research into entirely natural and biodegradable nanoemulsifier systems.

Despite their high pesticidal efficacy and plant growth‐promoting effects, the translation of green NE technologies from the laboratory to the field remains constrained by scalability, cost efficiency, and regulatory uncertainty, emphasizing the importance of integrated techno‐economic analyses and comprehensive toxicological assessments. One of the critical challenges for field application is the potential phytotoxicity of foliar‐applied formulations containing high concentrations of EOs (e.g., ≥ 5%). Therefore, future studies must establish safe concentration thresholds to ensure efficacy without harming plant tissues or compromising crop health, particularly in organic and low‐input farming systems.

Additionally, neglected targets, such as nematodes and plant viruses, represent promising avenues for innovation using nanoencapsulated phytochemicals. Future research should also investigate other nanoencapsulation strategies, including multiple emulsions (e.g., W/O/W and O/W/O systems), which may enhance the stability, loading efficiency, and controlled release of phytochemicals with diverse polarities, thereby improving field performance and long‐term applicability.

## Author Contributions

**Anna Paula Azevedo de Carvalho**: conceptualization, methodology, data curation, formal analysis, investigation, funding acquisition, project administration, writing – original draft. **David A. Weitz**: visualization, writing – review and editing. **Carlos Adam Conte‐Junior**: resources, funding acquisition, writing – review and editing.

## Funding

This work was funded by The Fulbright US Scholar Program 2021; Fundação Carlos Chagas Filho de Amparo à Pesquisa do Estado do Rio de Janeiro—Brasil (FAPERJ) [grants E‐26/200.621/2022, E‐26/210.385/2022, and E‐26/200.891/2021]; and the Conselho Nacional de Desenvolvimento Científico e Tecnológico—Brasil (CNPq) [grants 313119/2020‐1]. The funding sources have no role or involvement with this work.

## Ethics Statement

The authors have nothing to report. This study is based exclusively on previously published data and did not involve experiments with humans or animals.

## Conflicts of Interest

The authors declare no conflicts of interest.

## Data Availability

The datasets generated and analyzed during the current study are available from the corresponding author on reasonable request.
